# Branched actin networks mediate macrophage-dependent host-microbiota homeostasis

**DOI:** 10.1126/science.adr9571

**Published:** 2025-11-13

**Authors:** Luiz Ricardo C. Vasconcellos, Shaina Chor Mei Huang, Alejandro Suarez-Bonnet, Simon Priestnall, Probir Chakravarty, Sunita Varsani-Brown, Matthew L. Winder, Kathleen Shah, Naoko Kogata, Brigitta Stockinger, Michael Way

**Affiliations:** 1 Cellular Signalling and Cytoskeletal Function Laboratory, The Francis Crick Institute, 1 Midland Road, London, NW1 1AT, UK; 2 Experimental Histopathology, The Francis Crick Institute, 1 Midland Road, London, NW1 1AT, UK; 3 Department of Pathobiology and Population Sciences, Royal Veterinary College, Hatfield Hertfordshire, AL9 7TA, UK; 4 Bioinformatics and Biostatistics, The Francis Crick Institute, 1 Midland Road, London, NW1 1AT, UK; 5 Genetic Modification Service, The Francis Crick Institute, 1 Midland Road, London, NW1 1AT, UK; 6 AhR Immunity Laboratory, The Francis Crick Institute, 1 Midland Road, London, NW1 1AT, UK; 7 Department of Infectious Disease, Imperial College, London SW7 2AZ, UK

## Abstract

Branched actin networks formed by the Arp2/3 complex are essential for immune system function. Patients with loss-of-function mutations in the ARPC5 subunit of the Arp2/3 complex develop inflammation and immunodeficiency after birth, leading to early mortality. The basis for these phenotypes remains obscure. We found that loss of ARPC5, but not the ARPC5L isoform, in the mouse hematopoietic system caused early-onset intestinal inflammation after weaning. This condition was initiated by microbiota breaching the ileal mucosa and led to systemic inflammation. ARPC5-deficient macrophages and neutrophils infiltrated the ileum but failed to restrict microbial invasion. Specifically, macrophages lacking ARPC5 struggled to phagocytose and kill intra-cellular bacteria. Our results highlight the indispensable role of ARPC5-, but not ARPC5L-, containing, Arp2/3 complexes in mononuclear phagocyte function and host-microbiota homeostasis.

Mutations in genes encoding proteins involved in the regulation and organization of the actin cytoskeleton frequently result in primary immunodeficiency (1). These so-called actinopathies have a wide spectrum of severity involving the innate and/or the adaptive arm of the immune system (2). The most well-known actinopathy, Wiskott-Aldrich Syndrome (3), is caused by mutations in WASP, a hematopoietic specific protein that activates the Arp2/3 complex to nucleate branched actin filament networks (4, 5). A functioning immune system depends on these networks to drive cell migration, phagocytosis and immunological synapse assembly (6, 7). The Arp2/3 complex consists of seven evolutionarily conserved subunits: two actin-related proteins, ARP2 and ARP3, and five scaffolding subunits, ARPC1-ARPC5 (8, 9). However, in mammals three of the subunits have two different isoforms (ARP3/ARP3B, ARPC1A/ARPC1B and ARPC5/ARPC5L), each of which confers different properties to the Arp2/3 complex (10–12). The relevance of these subunit isoforms is evident from the observation that mutations in human ARPC1B result in severe immunodeficiency as well as impaired cytotoxic T lymphocyte maintenance and activity (13–16). More recently, loss-of-function mutations in ARPC5 have also been reported to result in gastrointestinal conditions and increased susceptibility to infection, leading to sepsis and early death (17, 18). The mechanistic basis for these clinical observations in patients with ARPC5 mutations remains unknown. We therefore examined the impact of the loss of ARPC5 in the mouse hematopoietic compartment to determine the basis for these immune phenotypes.

## Loss of ARPC5 in immune cells leads to spontaneous enteritis

**Fig. 1:**
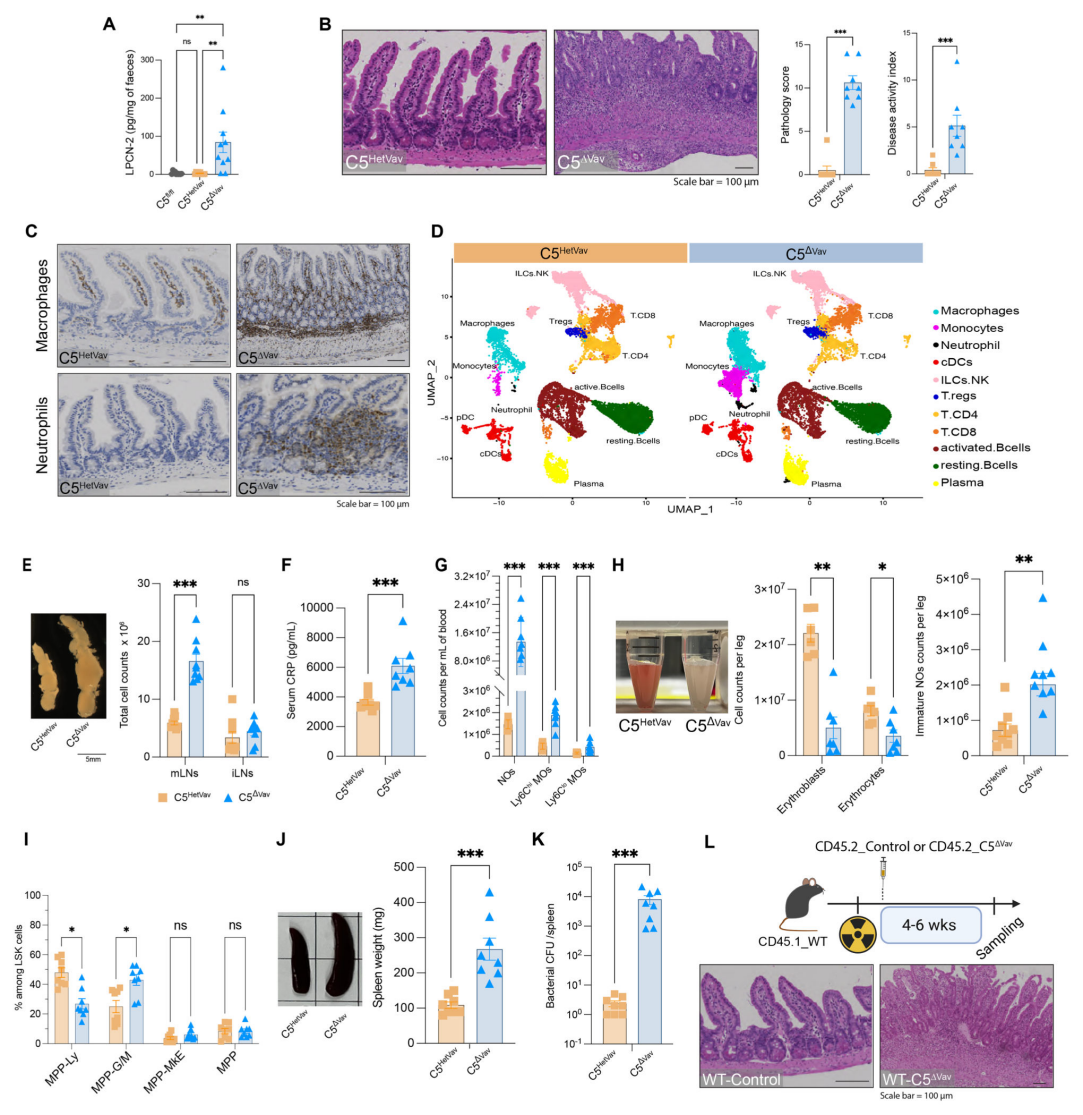
ARPC5 loss in immune cells leads to intestinal damage and systemic inflammation. (**A**) Lipocalin-2 (LPCN-2) faecal levels in the indicated mice. (**B**) H&E images of intestines (left) together with quantification of the histopathology score (middle) and disease activity index (DAI) (right). (**C**) Immunohistochemistry reveals the increased prevalence of macrophages (top) and neutrophils (bottom) in ileal sections in mice lacking Arpc5. **(D)** Uniform manifold approximation and projection (UMAP) of the integrated scRNAseq of 41130 cells from C5^HetVav^ or C5^ΔVav^ ileum lamina propria. Cell clusters are colored by the assigned cell cluster indicated in the figure. (**E**) Loss of Arpc5 increases the size and cell counts of mesenteric lymph nodes (mLNs) but not the inguinal lymph node (iLN). (**F-G**). Mice lacking Arpc5 have increased levels C-reactive protein (CRP) as well as phagocytes in their blood neutrophil (NO), monocytes (Ly6C^hi^MOs, Ly6C^lo^MOs). **(H)** Representative image of bone marrow together with the quantification of the counts of erythroid lineage and immature NO in the bone marrow of the indicated mice. (**I**) Quantification of the % of multipotent progenitors (MPP) among the lineage^-^Sca1^+^c-Kit^+^ (LSK) cells. MPP-Ly: lymphoid-biased MPPs; MPP-G/M: myeloid-biased MPPs; MPP-MkE: megakaryocyte and erythroid-biased MPPs; MPP: unbiased MPPs. (**J**) Representative image and weight of spleen in the indicated mice. (**K**) Bacterial colony forming units (CFU) in C5^HetVav^ or C5^ΔVav^ mice spleens. (**L**) Schematic of the bone marrow transplantation protocol: CD45.1_wild-type mice were lethally irradiated and reconstituted with either CD45.2_controls (WT-Control) or CD45.2_C5^ΔVav^ (WT-C5^ΔVav^) bone marrow (left). H&E images of intestines of animals which received wild-type (WT-control) or C5^ΔVav^ (WT-C5^ΔVav^) bone marrow transplantation (right). Representative gating strategies used in this figure are shown in figures S10-12. Animals assessed in A and L were 8-15 week old mice, n=10 of at least three experiments and n=7 of two independent experiments, respectively. For all the other panels, data shown of at least three independent experiments, n=8. In graphs, each data point represents data from an individual mouse, bars show the mean and error bars ± SEM. Data are pooled from at least three individual experiments. Statistical analysis was performed using Kruskal-Wallis test in A, Mann-Whitney test in B E, F, H, J, K, or multiple Mann-Whitney test in G and I. Significant *p* values are indicated on the graphs. C5^fl/fl^ (*arpc5*^fl/fl^), C5^HetVav^ (*arpc5*^HetVav^) and C5^ΔVav^(*arpc5*^ΔVav^). Scale bars = 100μm. ns = not significant. * = p-value < 0.05. ** = p-value < 0.01. *** = p-value < 0.001 and **** = p-value < 0.0001.

As ubiquitous loss of ARPC5 in mice is embryonic lethal (18), we used the Vav1-iCre driver (19), to delete *Arpc5* in the hematopoietic compartment (fig. S1A). At 8-15 weeks of age, *Arpc5*^fl/fl_Vav1Cre+^ animals (hereafter referred to as C5^ΔVav^) had reduced weight gain and increased levels of faecal lipocalin-2, a marker of intestinal inflammation (20), compared with littermate controls (C5^fl/fl^ and C5^HetVav^) (Fig.1A, fig. S1B). Consistent with this, adult C5^ΔVav^ had intestinal inflammation with increased infiltration of monocytes/macrophages and neutrophils, and loss of structural integrity, which was not seen in control mice (Fig.1B-C, fig. S1C-D). Single-cell RNA sequencing (scRNA-seq) analysis revealed that only monocytes or macrophages and neutrophils increased in the ileum of C5^ΔVav^ animals (Fig.1D). The inflammation and damage were restricted to the ileum of the small intestine (Fig. S1D-E). The mesenteric lymph nodes (mLNs) but not the inguinal lymph nodes (iLNs) were enlarged (Fig.1E), which is indicative of a local immune response in the intestine. The increase in size of mLNs was driven by an expansion of lymphocytes and recruitment of neutrophils and macrophages (fig. S1F). We did not observe inflammation or intestinal damage in age matched *Arpc5l*^fl/fl_Vav1Cre+^ (C5L^ΔVav^) animals (fig. S1G-H). We concluded that ARPC5, but not the alternative ARPC5L isoform, was essential for maintenance of intestinal homeostasis by the immune system.

## ARPC5 deficiency leads to systemic inflammation

Assessing the severity of inflammation, we found that C5^ΔVav^ mice had increased serum levels of C-reactive protein (CRP), a marker of inflammation (Fig.1F). The number of neutrophils and monocytes in the blood of C5^ΔVav^ mice increased, whereas only CD4 T cells decreased (Fig.1G and fig. S2A). Similar changes in cell numbers in the peripheral blood were reported in patients who lack ARPC5 (17). C5^ΔVav^ mice also had pale bone marrow due to a reduction in erythroid lineages and increased Ly6G^lo^ immature neutrophils (Fig.1H), as observed in mice with sepsis (21). Consistent with the increased myeloid cell counts, the proportion of myeloid-biased multipotent progenitors (MPP^G/M^) increased whereas lymphoid-biased ones (MPP^Ly^) decreased (Fig. 1I). Compared with controls, C5^ΔVav^ mice also had splenomegaly with increased numbers of neutrophils, macrophages, and dendritic cells (DCs), and reduced counts of natural killer cells (NKs) but no changes in B and T cells (Fig.1J, fig. S2B). Indicative of microbial dissemination, the spleens of C5^ΔVav^ animals contained more bacteria compared with those of the controls (Fig.1K). To determine whether the phenotype observed was cell intrinsic, we performed adoptive transfer of bone marrow in which ARPC5 was removed [*Arpc5* knockout (KO)] into wild-type mice that had been lethally irradiated to ablate their hematopoietic system (Fig.1L). These animals developed the pathology observed in C5^ΔVav^ mice, confirming that the phenotype was intrinsic to the hematopoietic compartment (Fig.1L and fig. S2C).

## The microbiota drives the inflammatory response in C5^ΔVav^ animals

**Fig. 2:**
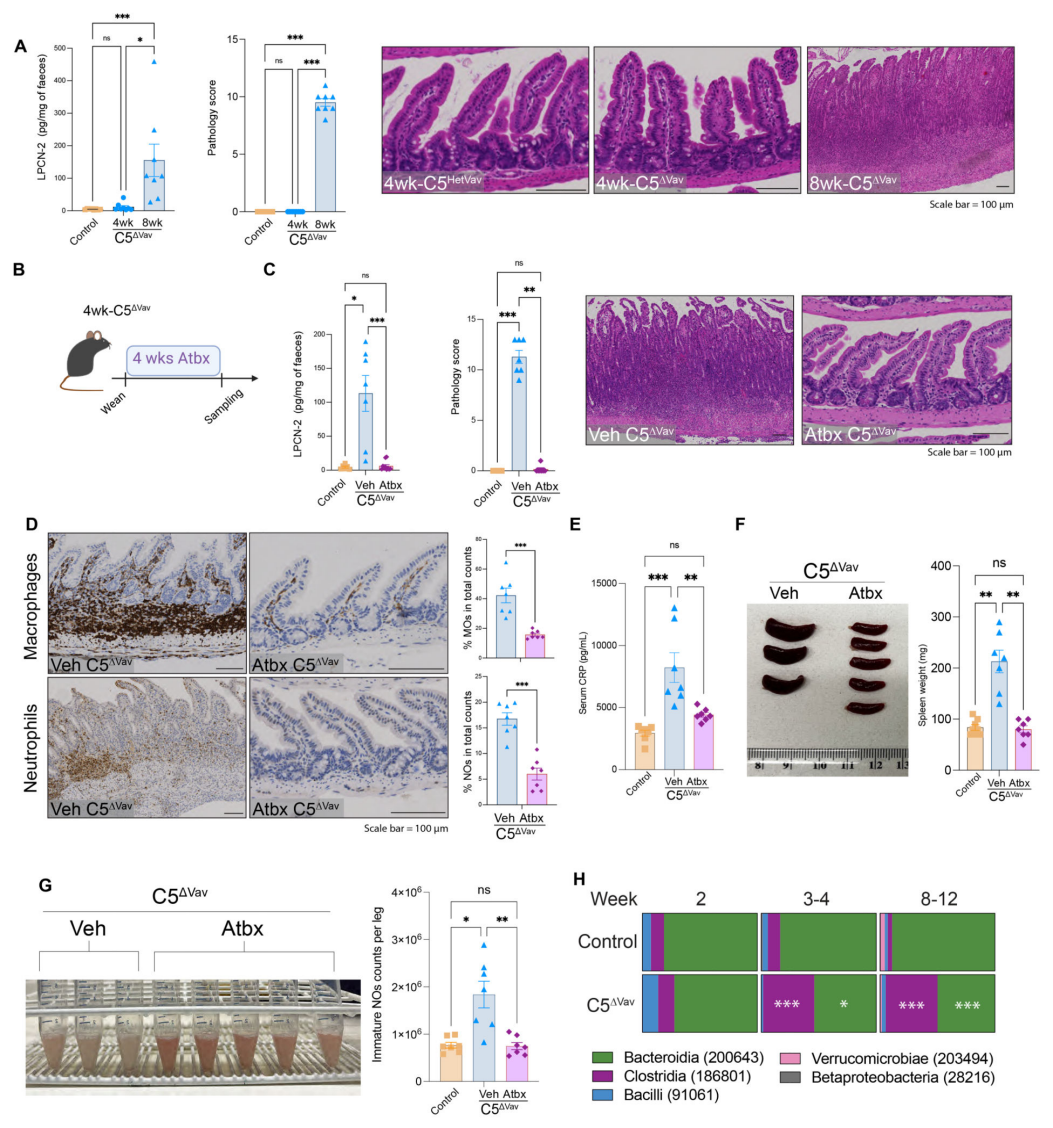
The intestinal dyshomeostasis upon ARPC5 loss is microbiota dependent. (**A**) Faecal lipocalin-2 (LPCN-2) levels (left), quantification of the histopathology score (middle) and H&E images of the ileum of 4-week-old (wk) C5^HetVav^ or 4 and 8wk C5^ΔVav^ animals (right). (**B**) Experimental scheme of antibiotic treatment; 4wk animals were treated with antibiotic cocktail (Atbx) or vehicle (Veh) for 4 weeks and then sampled for analysis. (**C**) LPCN-2 levels in faeces (left), and histopathology score (middle) together with H&E image of the ileum in the indicated animals (right). (**D**) Representative immunohistochemistry images and quantification showing Atbx treatment reduces macrophages (top) and neutrophils (bottom) presence in ileal sections in C5^ΔVav^ mice. **(E**) Levels of C-reactive protein (CRP) in the blood of animals with and without antibiotic treatment. **(F**) Representative image and weight of spleens in C5^ΔVav^ animals with and without antibiotics. (**G**) Representative image of bone marrow together with the quantification of numbers of immature neutrophil (NO) in the bone marrow of mice with and without antibiotic treatment. (**H**) Five most prevalent classes of bacteria found in the intestinal microbiome of control (C5^HetVav^) or C5^ΔVav^ animals at the indicated age (n=8 per group). Representative gating strategies used in this figure are shown in figures S10-12. In graphs, each data point represents data from an individual mouse, bars show the mean and error bars ± SEM (n=7). Data are pooled from at least three individual experiments. Statistical analysis was performed using Kruskal-Wallis tests in A, C, F and G, Mann-Whitney test in D, Multiple Mann-Whitney test in H, or One Way ANOVA in E. Significant *p* values are indicated on the graphs. Controls used were *Arpc5*^HetVav^. C5^ΔVav^ represents *Arpc5*^ΔVav^. Scale bars = 100μm. ns = not significant. * = p-value < 0.05. ** = p-value < 0.01. *** = p-value < 0.001 and **** = p-value < 0.0001.

At weaning there is a change in the intestinal microbiota that profoundly impacts mucosal homeostasis (22). To investigate the onset of pathologies induced by the absence of ARPC5, we assessed animals immediately after weaning (4 week old). We found that there was no increase in fecal lipocalin-2 levels, mLN enlargement, or intestinal pathology in 4-week-old C5^ΔVav^ animals compared with control littermates (Fig.2A, fig. S3A). Longitudinal RNA-seq analysis of intestinal macrophages from animals at 4, 6 and 8 weeks revealed that differentially expressed genes (DEG) pathways were mainly associated with inflammation, but only in adult mice (8 week old) (fig. S3B). However, the elevated numbers of macrophages and DCs in 4-week-old C5^ΔVav^ mLNs (fig. S3C) suggested that there was a progressive onset of inflammation after weaning. To explore whether local and systemic inflammation are triggered by commensal colonization after weaning, we treated 4-week-old C5^ΔVav^ animals with an antibiotic cocktail prior to development of enteritis (Fig. 2B). This treatment prevented the development of enteritis, infiltration of macrophages and neutrophils into the ileum, as well as subsequent systemic inflammation (Fig. 2C-E). Antibiotic treatment of C5^ΔVav^ mice also suppressed splenomegaly (Fig. 2F, fig. S4A) and enlargement of mLNs and maintained normal cell populations in the bone marrow (Fig. 2G, fig. S4B). Analysis of the intestinal microbiome revealed that the loss of ARPC5 in the immune system also changed the composition of the microbiota (Fig. 2H and fig. S4C). Even before the intestinal damage was evident, Clostridia and Bacteroidia classes increased and decreased, respectively, in 4-week-old C5^ΔVav^ animals (Fig. 2A and H). This change in microbiota composition was not seen before weaning (2week) (Fig. 2H and fig. S4C). We concluded that the onset of intestinal inflammation was progressive and driven by the microbiota.

## ARPC5 is essential for intestinal homeostasis

We next sought to determine whether the phenotype seen in C5^ΔVav^ mice was driven by a deficiency in innate and/or adaptive immune cells. Our scRNA-seq analyses of immune cells in the intestine demonstrated that innate immune cells increased in C5^ΔVav^ mice (monocytes or macrophages and neutrophils) (fig. S5A). Furthermore, DEG pathway analysis revealed increased transcription of genes associated with actin cytoskeleton dynamics, inflammation or innate immunity, extracellular matrix remodelling and oxidative stress (fig. S5B). By contrast, there was no difference in cell numbers in the adaptive compartment or impact on polyclonal activation in C5^ΔVav^ T cells isolated from mLNs (fig. S5, A and C). Similarly, no defect in T cell response was reported in patients with *ARPC5* mutations (17). Because an interaction between monocytes or macrophages and regulatory T cells (T_reg_ cells) in the intestine is essential for maintaining tolerance (23), we assessed the cell-to-cell cross-talk in our scRNA-seq dataset using CellChat (24). We found that there were increased differential interactions of monocytes or macrophages with themselves as well as with T_regs_ cells and other immune cells in the absence of ARPC5 (fig. S5D). Furthermore, T_reg_ cells in C5^ΔVav^ animals did not produce interleukin-10 (IL-10) (fig. S6A), which is required for a tolerogenic response (25). Consistent with the lack of IL-10 production, *Arpc5* KO T_reg_ cells failed to maintain intestinal homeostasis in a lymphocyte transfer model (fig. S6B).

**Fig. 3:**
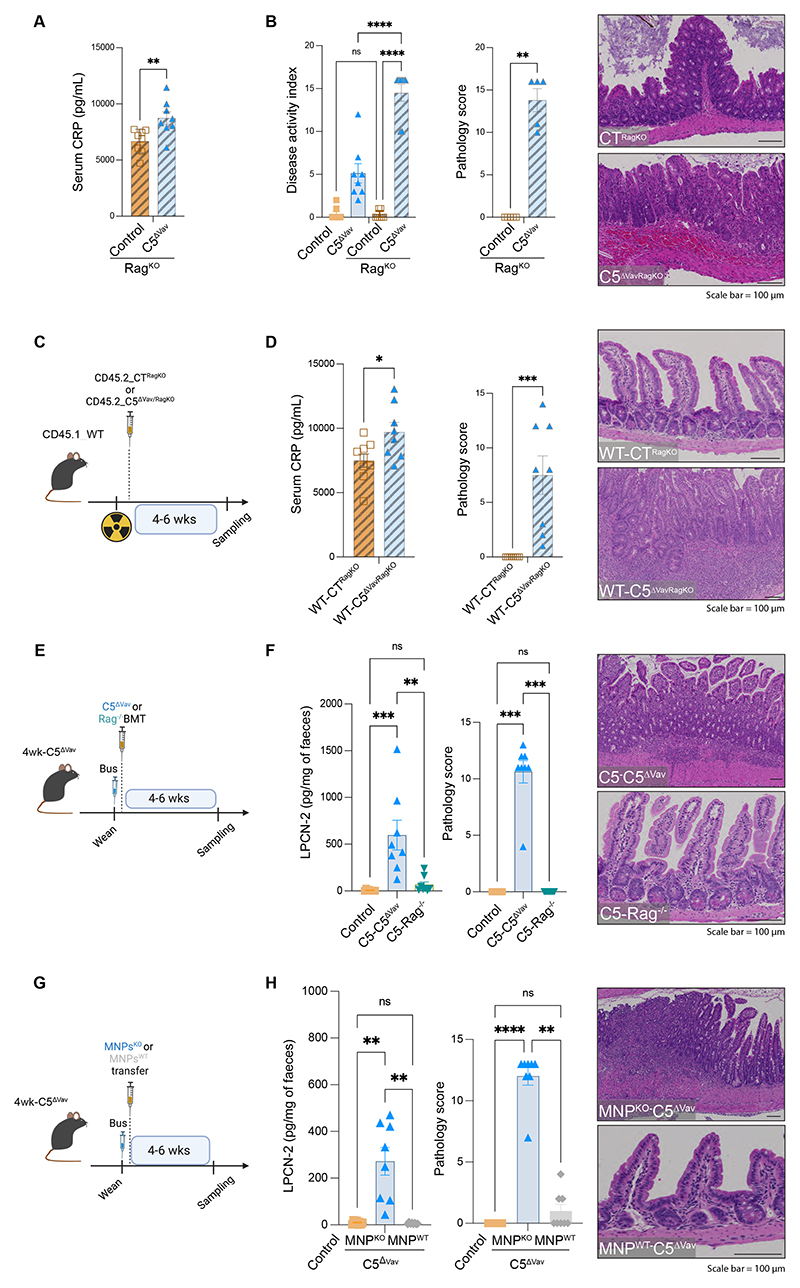
ARPC5 is essential for effective innate immunity. (**A**) Levels of C-reactive protein (CRP) in the serum and (**B**) quantification of disease activity index (DAI including values also shown in Fig.1C, left) and histopathology score together with representative H&E images of cecum of control^RagKO^ or C5^ΔVavRagKO^ mice (right). (**C**) Schematic of the bone marrow transplantation protocol: CD45.1_wild-type mice were subjected to lethal irradiation and reconstituted with either CD45.2_control^RagKO^ (WT-CT^RagKO^) or CD45.2_C5^ΔVavRagKO^ (WT-C5^ΔVavRagKO^) bone marrow. (**D**) Levels of CRP (left) and quantification of the histopathology score in irradiated mice together with representative H&E images of ileum (right). (**E**) Scheme for Rag^-/-^ adoptive cell transfer after busulfan treatment: 4-week-old (wk) C5^ΔVav^ mice were treated with busulfan and injected with either WT (C5-Rag^-/-^) or C5^ΔVav^ (C5-C5^ΔVav^) innate cells. (**F**) Lipocalin-2 (LPCN-2) faecal levels (left) and quantification of the histopathology score together with representative H&E images of the ileum of busulfan-treated mice (right). (**G**) Scheme for mononuclear phagocytes (MNPs) adoptive transfer into 4 wk C5^ΔVav^ busulfan depleted mice: animals were transplanted with either wild-type (MNP^WT^) or C5^ΔVav^ (MNP^KO^) cells. (**H**) LPCN-2 faecal levels (left) and quantification of the histopathology score together with representative H&E images of the ileum (right) of indicated animals. In graphs, each data point represents data from an individual mouse, bars show the mean and error bars ± SEM (n=8). Data are pooled from at least three individual experiments. Statistical analysis was performed using Mann-Whitney test in A, B (right) and D; one-way ANOVA test in B (left) and Kruskal-Wallis tests in F and H. Significant *p* values are indicated on the graphs. Controls used were *Arpc5*^HetVav/RagKO^. C5^ΔVavRagKO^ represents *Arpc5*^ΔVav/RagKO^. Scale bars = 100μm. ns = not significant. * = p-value < 0.05. ** = p-value < 0.01. *** = p-value < 0.001 and **** = p-value < 0.0001.

To investigate the contribution of the adaptive immune system in the intestinal phenotype induced by the absence of ARPC5, we crossed C5^ΔVav^ mice with *Rag^-/-^* mice (C5^ΔVav/RagKO^), which lack T and B cells. C5^ΔVav/RagKO^ mice had an even more pronounced phenotype, with all animals reaching an humane end point around 6 weeks of life because of intestinal and systemic inflammation (Fig.3A-B and fig. S7A). In these C5^ΔVav/RagKO^ mice, the intestinal damage was now also observed in the cecum (fig. S7A). In addition, we observed that there were similar changes in the microbiota of C5^ΔVav/RagKO^ animals as in the C5^ΔVav^ mice (fig. S7B). Adoptive transfer of C5^ΔVav/RagKO^ bone marrow into lethally irradiated wild-type mice also transferred the pathological phenotype (Fig.3, C and D and fig. S7, C and D). This confirmed that the phenotypes seen in C5^ΔVav^ mice were driven by deficiencies in innate immunity.

The current treatment for Wiskott-Aldrich Syndrome involves transplantation of hematopoietic stem and/or progenitor cells (26, 27). We decided to use a similar strategy, performing adoptive transfer of innate immune cells derived from *Rag*^-/-^ bone marrow into 4-week-old C5^ΔVav^ animals after myeloablation (Fig. 3E). We found that C5^ΔVav^ animals reconstituted with wild-type cells (C5-Rag^KO^) had no signs of inflammation or the pathologies seen in C5^ΔVav^ mice (Fig. 3F, fig. S8, A and B).

Mononuclear phagocytes (MNPs) play a key role in safeguarding intestinal homeostasis by clearing microbes that breach the ileal mucosa (28–31). Given this, we performed adoptive transfer of CD115^+^ MNPs from wild-type or C5^ΔVav^ bone marrow into myeloablated 4-week-old C5^ΔVav^animals (MNPs^WT^-C5^ΔVav^ and MNPs^KO^-C5^ΔVav^, respectively) (Fig. 3G). MNPs^WT^-C5^ΔVav^ animals developed no enteritis or splenomegaly and had levels of circulating CRP and fecal lipocalin-2 comparable with those of controls (Fig. 3H and fig. S8C). Similarly, wild-type MNPs transfer after peripheral depletion with clodronate liposomes ameliorated the pathology in C5^ΔVav^ animals, although inflammation was still present (fig. S8D). We concluded that innate immune cells, and more specifically macrophages, promoted the inflammatory phenotypes observed in the absence of ARPC5.

## ARPC5 is required for efficient phagocytosis and bacterial killing

**Fig. 4:**
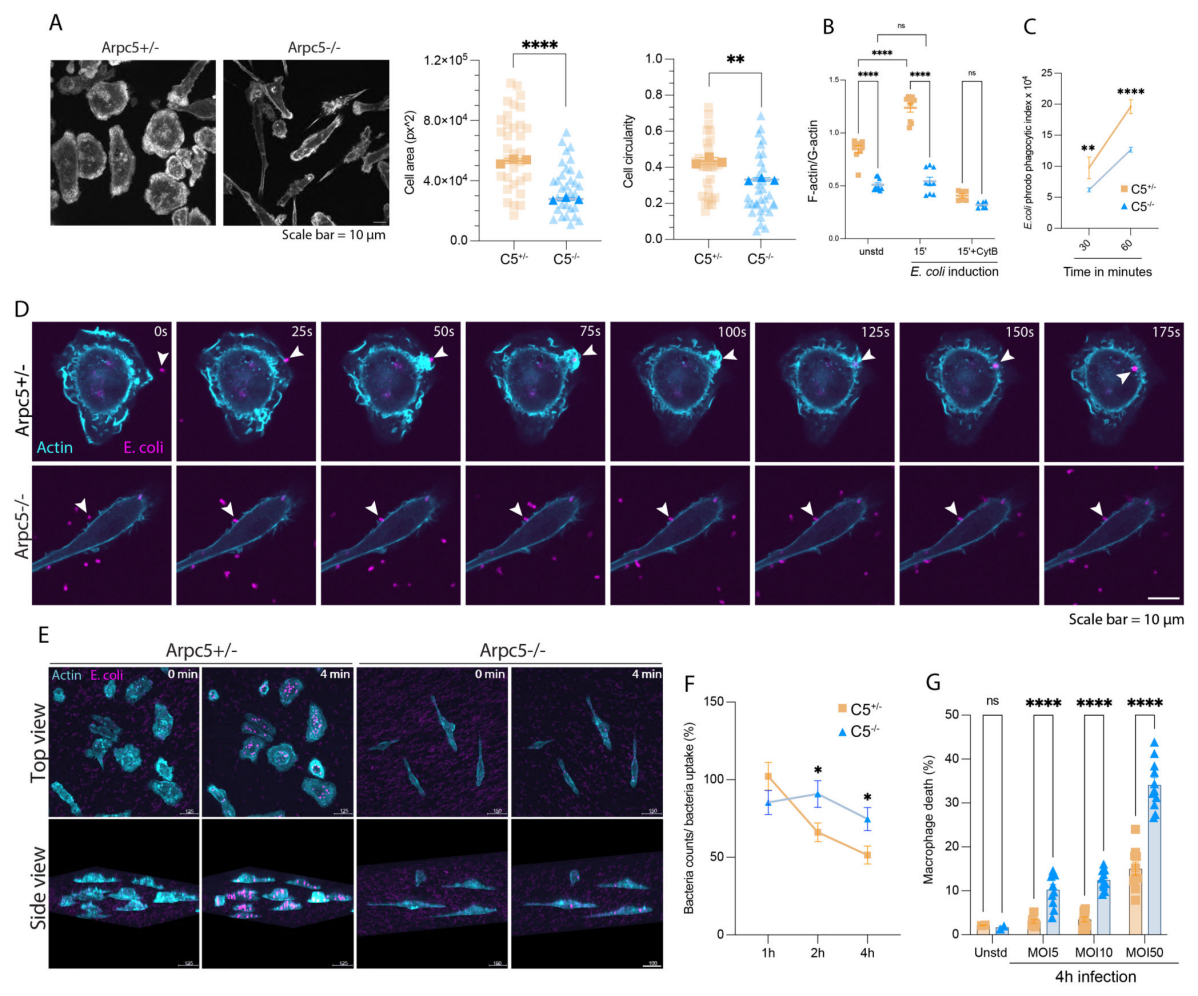
ARPC5-mediated actin remodeling is key to macrophage biology. **(A)** Representative images of the actin cytosksleton in bone marrow derived macrophages (BMDMs) with or without ARPC5 together with quantification of their cell area and circularity. Three independent experiments with 10-12 cells measured per experiment. (**B**) Ratio of F-actin/G-actin in resting or *E.coli*-activated BMDMs incubated with vehicle or pretreated with 10μM Cytochalasin D for 30min. Each data point represent data from a technical replicate, bars show the mean and error bars ± SEM. Data are representative of three individual experiments. (**C**) Quantification of *E. coli* phrodo particle uptake by BMDMs. Bars show the mean and error bars ± SEM. Data are representative of three individual experiments. (**D**) Representative images showing the actin cytoskeleton dynamics (Cyan) visualized with GFP-Lifeact in Arpc5^+/-^ or Arpc5^-/-^ BMDMs during *E. coli* (Magenta) phagocytosis. The time in each panel is seconds. (**E**) Representative images showing top and side views (3D representation) of *E. coli* (Magenta) in BMDMs. Scale bar (μm) are indicated in each panel. (**F**) The quantification of the percentage of internalized bacteria by BMDMs. Bars show the mean and error bars ± SEM. Data are representative of three individual experiments. (**G**) *E. coli* infection induced BMDM cell death. Each data point represent data from a technical replicate, bars show the mean and error bars ± SEM. Data are representative of three individual experiments. Statistical analysis was performed using unpaired *t* test in A; two-way analysis of variance (ANOVA) in B, C and F and multiple Mann-Whitney test in G. Significant *p* values are indicated on the graphs. C5^+/-^ and C5^-/-^ represent *Arpc5*^+/-^ and *Arpc5*^-/-^ respectively. Scale bars = 10μm. ns = not significant. * = p-value < 0.05. ** = p-value < 0.01. and **** = p-value < 0.0001.

ARPC5 deficient bone marrow-derived macrophages (BMDMs) had a distinctive cell morphology, being less spread and more elongated than wild-type counterparts (Fig.4A, fig. S9A). These differences indicated that loss of ARPC5 affected the organization of the actin cytoskeleton as reported in other cell types (17, 18). Our histological analysis, however, indicated that macrophages without ARPC5 readily infiltrate into the small intestine (Fig 1C), suggesting that defects in cell migration were not a major contributing factor in the phenotype of C5^ΔVav^ mice. Nevertheless, BMDMs that lack ARPC5 had notably reduced levels of F-actin indicative of altered actin dynamics (Fig. 4B, fig. S9B).

It is well established that microbial internalization by phagocytes is actin dependent, and defects in this process may contribute to inflammatory bowel disease (IBD) (32, 33). Consistent with this, we found that BMDMs lack ARPC5 were less efficient than controls in phagocytosing *Escherichia coli* and were defective in actin filament assembly (Fig.4, B and C and fig. S9, C and D). Live cell imaging of BMDMs expressing green fluorescent protein (GFP)-LifeAct revealed that the absence of ARPC5 resulted in a loss of actin-rich ruffles required for bacterial phagocytosis, and this correlated with a decrease in their ability to engulf particles (Fig 4, D and E, and movies S1 and S2). Furthermore, in contrast to wild-type BMDMs, the loss of ARPC5 also led to reduced bacterial killing because the number of intracellular bacteria remained constant (Fig. 4F). Consistent with their defective bactericidal activity, infected BMDMs lack ARPC5 were also more prone to die (Fig. 4G). Our analyses supported the notion that ARPC5-deficient macrophages were defective in phagocytosing and killing microbes that breach the intestinal barrier.

## Discussion

The Arp2/3 complex induces the assembly of branched actin networks that drive cell migration, phagocytosis, and immune synapse formation that a normal functioning immune system requires (7, 34, 35). Mutations in both the upstream activators of the Arp2/3 complex and the complex itself can lead to immunodeficiency and inflammation (13–16, 36, 37). More recently, we and others have found that loss of the ARPC5 subunit of the Arp2/3 complex results in intestinal inflammation, recurrent infections, and sepsis, leading to early mortality (17, 18). We developed a mouse model that has enabled insights into immune symptoms in patients who lack ARPC5.

In our model, the intestinal pathology associated with ARPC5 deficiency in hematopoetic cells was restricted to the small intestine (ileum) but not the large instestine, in contrast to what has been reported in a mouse model of Wiskott-Aldrich Syndrome (38, 39). In our model, the inflammation in the ileum was associated with changes to the microbiome upon weaning. The lack of inflammation in the large intestine is likely because the protective double mucus layer prevents the altered microbiome from reaching the colonic epithelium (40, 41). Moreover, this restriction could be explained by the mice being housed in a facility where pathobionts, including *Helicobacter* sp., that induce large intestine pathologies were absent. Animal welfare considerations prevented us from assessing whether the inflammation and tissue pathology seen in the absence of ARPC5 extended from the ileum to other region of the intestine in older animals.

We observed an increase in both monocytes or macrophages and neutrophils in the ileum of inflamed *Arpc5* KO animals. Although infiltration of neutrophils likely contributes to the overall intestinal pathology, transplantation of wild-type monocyte or macrophage precursors was sufficient to revert the intestinal pathology of the ARPC5-deficient animals. This indicated that pathology is driven by the inability of macrophages to maintain the intestinal barrier. Moreover, the pathology of ARPC5 deficiency was not dependent on migration of macrophages to the intestine but rather their inability to perform phagocytosis and microbial killing. The Arp2/3 complex is also not required for DCs to migrate to the intestinal lamina propria (42), but a functional Arp2/3 complex is required for macrophage phagocytosis (35, 43–45).

Although the inflammatory effect observed in C5^ΔVav^ animals does not require the adaptive immune system, it is likely to be exacerbated by defective T_reg_ cells that cannot produce IL-10. ARPC5-dependent innate immunity also regulated the composition of the intestinal microbiota before to the onset of intestinal inflammation. Further work will be required to understand how ARPC5-dependent actin polymerization in innate immune cells regulates the composition of the microbiota. We used antibiotic treatments to disrupt the colonization of microbiota in the gut, but examination of animals in germ-free conditions might provide additional insights, including understanding roles of ARPC5 in immune system homeostasis that are independent of the microbial colonization.

Our study explains why patients who lack ARPC5 develop gastrointestinal complications and immunodeficiency and are prone to fatal sepsis. We propose that mutations in *ARPC5* may contibute to IBD and should be considered in patients with mutations in other candidate genes, such as *ARPC1B* and *WAS* (46, 47). Last, our results suggest that bone marrow transplantation would revert the immunodeficiency of patients with ARPC5 loss-of-function mutations.

## Supplementary Material

fig. S1

movie S1

movie S2

## Data Availability

The scRNA-seq and bulkRNA-seq data associated with this study are publically available at the NCBI GEO repository with the identifiers GSE298330 and GSE298655, respectively. The raw microbiome data associated with this study are publically available at the NCBI Sequence Read Archive (SRA) repository with the identifier PRJNA1309310. All other data needed to evaluate the conclusions in the paper are available in the main text or the supplementary materials. The *Arpc5* and *Arpc5l* conditional knockout mice on a C57Bl/6 background that are described in this study are available from M.W. under a material agreement with The Francis Crick Institute.
