# Supplementary material for: Branched actin networks mediate macrophage-dependent host-microbiota homeostasis
Source: Science. Author manuscript; Available in PMC 2025 Nov 24. (PMC7618398; doi:10.1126/science.adr9571)
Supplement: fig. S1 [file EMS210861-supplement-fig__S1.pdf]

Science AAAS

Supplementary Materials for

Branched actin networks mediate macrophage-dependent host-microbiota homeostasis

Luiz Ricardo C. Vasconcellos *et al*.

Corresponding author: Michael Way, michael.way@crick.ac.uk

Science **390**, 728 (2025)
DOI: 10.1126/science.adr9571

The PDF file includes:

Materials and Methods
Figs. S1 to S13
References

Other Supplementary Material for this manuscript includes the following:

MDAR Reproducibility Checklist
Movies S1 and S2

Materials and Methods

Mice

In the present study we utilized sex and age-matched (4-15 weeks old) C57Bl/6 mice, from both genders, bred at the Francis Crick Institute (2019-2024) under pathogen-free conditions. Mice were held in individual cages with a maximum of five animals. Animals were maintained within IVC green line system with air movement regulated by air handling unit on negative pressure (75 ACH/-20%) (Techniplast, UK). The animals were kept under a 12h light/dark cycle (7am-7pm including dawn and dusk settings of 15 min) and room temperature and humidity of 22°C (+/- 2°C) and 55% (+/-10%), respectively. The animals had *ad libitum* access to water (chlorinated to 2.45%) and food (Teklad Global Rodent Diet Sterilised 2018S -18% protein) (Inotiv, Indiana) and were kept on Eco Pure Chips sawdust, Bed'r'Nest nesting and smart homes enrichment (Datesand, UK). For tissue collection, mice were euthanized by schedule 1 procedure in accordance with national and institutional guidelines for animal care. All in vivo experiments were performed following the Act 1986 for animal scientific procedures after the approval by the review board of The Francis Crick Institute and the Home Office (United Kingdom) under project licence PP0792028. Mice were genotyped before weaning by Transnetyx and randomly assigned to treatment groups and blinding strategies applied when possible. For adoptive transplantation, both *Ptprc*^{a} (CD45.1) and *Ptprc*^{b} (CD45.2) were used. The genetically modified animals were bred on a C57Bl/6 background and the *Arpc5*$^{\text{Cre-Vav1}}$, *Arpc5*$^{\text{Cre-Foxp3-YFP}}$*, Rag2*$^{-/-}$, *Rag1*$^{-/-}$, *Rag1*$^{-/-}$/*Arpc5*$^{\text{Cre-Vav1}}$, C57Bl/6$^{\text{LifeAct-GFP}}$ and *Arpc5*$^{\text{Cre-Vav1/ LifeAct-GFP}}$ (*48*) were used in the present study.

The conditional Arpc5 knockout mouse model has been previously reported (*18*). The Arpc5l conditional knockout was generated by the Crick Genetic Modification Service using a similar CRISPR-Cas9 approach in embryonic stem cells (ESCs). Briefly, the gRNA-1: 5'-CTCTGGATAGAGACAACAGA-3' and gRNA-2: 5'-CATCCCCGATAGAGCTACAT-3' were used to introduce the 5′ and 3′ loxP sites flanking exon 2 of the Arpc5l gene. Deletion of exon 2 results in a frameshift and an exogenous stop codon within exon 3. The Cas9-gRNA-Puro plasmids were generated by inserting the CRISPR-Cas9 target sequences into PX459 plasmid (Addgene plasmid #48139). The pMA donor plasmid contained a synthesized 1049 bp fragment corresponding to exon 2 flanked by loxP sites and 1 kb flanking homology arms (GeneArt, Thermo Fisher Scientific). Sequence-verified Cas9-gRNA-Puro plasmids and pMA donor plasmid were co-transfected into in-house C57BL6/N 6.0 ESC using Lipofectamine 2000 (Thermo Fisher Scientific) and selected with puromycin. Integration of 5′ and 3′ loxP sites were identified by reverse transcription quantitative polymerase chain reaction (RT-PCR) assays on genomic DNA using gene-specific probes designed by Transnetyx and the integrity of the loxP integrations was confirmed by PCR amplicons sequencing. ESC clones were microinjected into C57BL/6J mice blastocysts [B6(Cg)-Tyrc-2J/J; strain #000058, The Jackson Laboratory) and then transplanted into the uteri of pseudo-pregnant CD1 females. The resulting chimeras were crossed to albino C57BL/6J, and their offspring screened for the integration of the loxP sites by amplicon sequencing. Heterozygous floxed offspring were validated by sequencing and two founder strains maintained on a C57BL/6J background.

Disease activity index and histology

To assess the enteritis disease activity index (DAI), we followed a modified scoring system as described previously (*49*). Briefly, mice were evaluated using signs of clinical disease including weight loss, faecal consistency and presence of blood, and lack of movement. All parameters were

assessed using a semi-quantitative grading scheme as follows: 0, no disease; 1, mild; 2, moderate; 3, marked; 4, severe.

Intestines ('Swiss rolls') and other organs were collected, fixed for 48h in 10% neutral buffered formalin which was subsequently replaced with 70% ethanol, embedded in paraffin wax and sectioned at 4µm. Sections were stained with haematoxylin and eosin (H&E) then microscopically examined by two certified veterinary pathologists blinded to the grouping and genotypes. Histopathological analyses considered the presence and extent of tissue inflammation and epithelial injury. All parameters were assessed in a semi-quantitative grading scheme for severity as follows: 0, no lesion; 1, minimal change; 2, mild change; 3, moderate change; 4, marked change; 5, severe.

Quantification of total leukocytes, macrophages and neutrophils immunohistochemistry was performed using anti-CD45, anti-F4/80 or anti-2B10, respectively, conjugated with a horseradish peroxidase (HRP) secondary antibody and using DAB chromogen. The slides were scanned with a Zeiss Axio Scan.Z1 annotated and processed with QuPath software (v0.4.3). To account for the prevalence of target cells, 10 different random regions (750µm^2) were analyzed for positive labelling using hematoxylin as counterstaining. The values are presented as prevalence (%) of the Target cell/ Total cells.

Irradiated chimeras

CD45.1$^+$ wild-type recipient mice at 10-13 weeks old were lethally irradiated with ^{137}Cs-irradiated 2 × 6 Gy with a 3-hour interval. For reconstitution, 10 x 10^6 bone marrow cells from donors (CD45.2$^+$, as indicated in figures) were injected intravenously via tail vein into the recipients (CD45.1$^+$). Bone marrow cells were collected from femur and tibia of donor mice and washed through a 70µm cell strainer. At 5-10 weeks later, the animals were euthanized, samples collected, and engraftment verified by flow cytometry. Distinction between donor and recipient cells was assessed by CD45.1 or CD45.2 expression.

Antibiotic treatment

Four week old *Arpc5*$^{\text{Cre-Vav1}}$ (C5$^{\Delta\text{Vav}}$) mice were treated continuously for 4 weeks in the drinking water with an antibiotic cocktail (Atbx) consisting of ampicilin (1mg/mL), gentamicin (1mg/mL), metronidazole (1mg/mL), neomycin (1mg/mL), vancomycin (500µg/mL) and sucralose-based artificial sweetener (100mg/mL, The Pantry) or artificial sweetener only (vehicle). Mice were euthanized, and samples collected after treatment.

Intestinal microbiome sequencing and analysis

Fresh faecal samples were collected from individual animals using the Microbiome collection kit (Transnetyx, Cordova, USA) and shipped to Transnetyx for DNA extraction using the Qiagen DNeasy 96 PowerSoil Pro QIAcube HT extraction kit (Qiagen, #47021). After DNA extraction and quality control, genomic DNA was converted into sequencing libraries using the KAPA HyperPlus library preparation protocol (Roche, 07962401001). Libraries were sequenced using the shotgun sequencing (a depth of 2 million 2x150 bp read pairs), using the Illumina NovaSeq instrument and protocol (Illumina, 20068232). Data were uploaded automatically onto One Codex analysis software (Wilmington, USA) and analyzed against the One Codex database of microbial reference genomes.

Busulfan chimeras

Four-week-old *Arpc5*$^{Cre\text{-}Vav1}$ (C5$^{\Delta Vav}$) mice were treated with 10mg/kg Busilvex (busulfan-Pierre Fabre) intraperitoneally for 2 consecutive days with a 24h interval. All donor bone marrow cells (10 x 10^6) or 5 x 10^6 CD115$^+$ (isolated following the manufacturer's instructions - Miltenyi Biotec, #130-096-354) in 100µl PBS were injected intravenously into the tail vein, 24h after the second busulfan dose (*50*). Bone marrow cells were collected from femur and tibia of donor mice and washed through a 70µm cell strainer. After adoptive cell transfer, animals were kept for 8-10 weeks before euthanasia and sample collection. CD45.1 and CD45.2 expression were used to differentiate between donor and recipient cells in transplanted animals.

In vivo macrophage depletion and adoptive cell transfer

Four-week-old *Arpc5*$^{Cre\text{-}Vav1}$ (C5$^{\Delta Vav}$) mice received intraperitoneal injection of 5µl/g clodronate_SUV_PEG liposomes (CP-SUV-P-005-005-Liposoma) and 48h later were adoptively transferred with 3 x 10^6 CD115$^+$ isolated from wild-type or C5$^{\Delta Vav}$ mice bone marrows following the manufacturer's instructions (Miltenyi Biotec, #130-096-354).

Immunoblot and Enzyme-linked immunosorbent assay (ELISA)

Cells and tissue extracts were lysed for 15 min on ice in RIPA buffer supplemented with protease and phosphatase inhibitors (Roche, catalogue number). Protein extracts were quantified, and similar amounts of total cell lysate separated by SDS-PAGE, followed by immunoblot analysis. Antibodies used for immunoblots: ArpC5 (Synaptic Systems, 305011), ArpC5L (Proteintech, 22025-a-AP), β-actin (Abcam, ab179467), anti-mouse IgG-IRDye® 800CW (LI-COR Biosciences, 926-32210) and anti-rabbit IgG-IRDye® 680RD (LI-COR Biosciences, 926-68071).

For LPCN-2 assessment, fresh faeces pellets were obtained and weighed prior to maceration in 300µl of PBS 0.1% triton X100 (Sigma) followed by ELISA detection. The levels of Lipocalin-2 (LPCN-2, DY1857-05) and C-reactive protein (CRP, MCRP00) were detected following the manufacturer's instructions (R&D Systems).

Splenic Colony forming units (CFU)

Mouse spleens were dissociated in 500µl PBS 0.2% NP40 (Merck-492016) and centrifuged at 400x*g* for 3 min at room temperature to pellet debris. 100µl of the supernatant was plated on LB agar, incubated at 37ºC for 24h and CFU determined.

Flow cytometry

Single cell suspension was preincubated with viability dye and FcgRIII/II (Fc block) before 30 min of incubation with fluorochrome-labelled antibodies or phalloidin. The stained cells were analyzed using BD LSR Fortessa cell analyser (BD Biosciences) and interpreted using Flowjo software (v.10.6.2). For intracellular staining cells were fixed and permeabilized using the Foxp3/Transcription Factor Staining Buffer Set (00-5523-00 ThermoFisher Scientific). FACs buffer (Thermo Fisher Scientific, 00-4222-26), Live/Dead™ Fixable Near-IR Dead Cell Stain Kit (Thermo Fisher Scientific, L10119).

The following antibodies (supplied by Biolegend or the indicated company) were used in flow cytometry: CD16/32 Fcx (101320), B220-FITC (103205), B220-PerCP (103234), B220-APC (103212), CD2-FITC (100105), CD3-BV711 (100241), CD3-BUV395 (BD, 563565), CD4-

BV605 (100548), CD4-BV711 (100557), CD8a-BV650 (100742), CD8a-BV737 (BD, 612759), CD8a-PE/Cy7 (100722), CD8b-BV786 (BD, 740952), CD11b-BV421 (101236), CD11b-BV711 (101242), CD11b-BV785 (101243), CD11c-AF488 (117311), CD11c-BV605 (117334), CD11c-BUV563 (BD, 749040), CD19-BV711 (115555), CD16/32-BV711 (101337), CD19-APC/Cy7 (BD, 557655), CD45-APC (103112), CD45-BUV395 (BD, 564279), CD45.1-AF700 (110724), CD45.2-BV785 (109839), CD48-PE (103406), CD64-PeCy7 (139314), CD71-PE (113807), CD103-AF488 (121408), CD103-BV605 (BD, 740355), CD117-APC (BD, 553356), CD135-BV421 (BD, 562898), CD150-BV605 (115927), F4/80 (Invitrogen, 17-4801-82), Foxp3-PE (126404), Foxp3-AF700 (126422), Gr-1-FITC (108406), Gr-1-BV711 (108443), I-A/I-E-AF488 (107616), I-A/I-E-AF700 (107622), Ly6C-BV785 (128041), Ly6C-APC (128016), Ly6G-FITC (BD, 551460), CD44-BV421 (103040), CD69-FITC (104506), NK1.1-BV711(108745), NK1.1-PE (BD, 557391), TCRbeta-BV421 (109230), TCRgd-APC (118116), TCRbeta-PE (eBioscience, 12-5961-82), Ter119-FITC (116206), Sca-1-PE/Cy7 (Invitrogen, 25-5981-82), AntiDNAse-I (Invitrogen, D12371) and $CD45^{rb}$-AF647 (562848).

Leukocytes isolation from intestinal lamina propria

Leukocytes were isolated from mice intestines following an adapted protocol provided by Miltenyi Biotec (Lamina Propria Dissociation Kit, 130-097-410). Small intestines were isolated, and residual fat tissue and Peyer's patches removed. The faeces were cleared and the tissue opened longitudinally in cold 10mM Hepes-PBS without Ca^{+2} and Mg^{+2} (PBSCM). Tissue was cut laterally into 0.5 cm length pieces and washed twice with predigestion solution (1mM DTT, 5mM EDTA, 5% FCS in PBSCM) for 20 min at 37ºC with rotation. Samples were vortexed for 10 sec, filtered and incubated with rotation for 20 min at 37ºC in PBSCM prior to being vortexed for 10 sec and filtered with a 100µm cell strainer. The strained samples were transferred to gentle MACS C Tube (Miltenyi Biotec, 130-093-237) containing pre-heated digestion solution (50µl Enzyme D, 50µl Enzyme R, 6.25µl Enzyme A) and digested in the program 37_m_LPDK_1 using the MACS Octo Dissociator with Heater (Miltenyi Biotec, 130-134-029). After digestion, samples were resuspended in FACS buffer (Thermo Fisher, 00-4222-26) and filtered through a 100µm cell strainer, cells counted with a Neubauer chamber and resuspended in appropriate volume for further assessment.

RNA-seq and analysis of ileum lamina propria macrophages

Macrophages were FACS sorted from ileum lamina propria of individual mice using a gating strategy to collect live cells, $lineage^-$ (CD3/CD19/NK1.1/Ly6G) and $CD45^+CD11b^+CD64^+$. RNA was extracted from samples using the QIAShredder and RNeasy Mini Kit with on-column DNase digestion, following manufacturer's instructions (Qiagen, 74904). RNA-seq libraries were made using total RNA with KAPA RNA HyperPrep with RiboErase following manufacturer's instructions (Roche, 08098131702). SMART-Seq libraries were pooled and sequenced on an Illumina NovaSeq 6000 (2 × 100 bp) (Illumina). Demultiplexed FASTQ files yielded a median 32.4 million paired reads per sample (IQR 4.7 M). All processing followed the nf-core/rnaseq pipeline v3.18.0 executed under Nextflow v24.04.2 with Singularity v3.6.4 on a high-performance cluster. Adapters and low-quality bases were removed with TrimGalore v0.6.10 (Cutadapt v4.9, FastQC v0.12.1). Post-QC, ≥ 98 % of bases per sample retained a Phred ≥ 30. Pipeline quality metrics from FastQC, Qualimap v2.3, RSeQC v5.0.2 and Samtools v1.21 were aggregated with MultiQC v1.18. Reference indices were built from the Mus musculus genome (GRCm38, Ensembl 95). Reads were mapped with STAR v2.7.11b in two-pass mode and simultaneously

quantified in selective-alignment mode with Salmon v1.10.3. Transcript-level abundances were summarized to length-scaled gene-level counts with tximport v1.28.0; genes with < 10 raw counts in < 50 % of samples were excluded (edgeR filterByExpr, edgeR v3.42.0). Count matrices were imported into DESeq2 v1.38.3. Size-factors used the median-ratio method; dispersions were fitted to a log-normal prior. Variance-stabilised counts (vst) informed PCA, UMAP (umap v0.2.10.0) and distance heat-maps. Differential expression: a composite factor group (KOvsCTsgenotype × developmental stage) encoded six biological states. Global comparisons comprised: (i) within-genotype time-courses and (ii) genotypes at each developmental stage. Log_2-fold-changes were shrunk with an adaptive-shrinkage prior (ashr v2.2-54) and genes with Benjamini–Hochberg FDR < 0.05 were deemed significant. Time-dependent genes were detected via a likelihood-ratio test (LRT) contrasting full (genotype + developmental stage) against reduced (genotype) models. Pathway & gene-set enrichment: single-sample enrichment scores were computed from vst matrices using ssGSEA (GSVA v1.48.0) for MSigDB Hallmark (H) and KEGG (C2) collections obtained via msigdbr v7.5.1. Scores were analyzed with limma v3.56.2 using the same contrast matrix, with FDR < 0.05 marking significance. Complementary preranked GSEA (clusterProfiler v4.6.2) used signed log_2-fold-change lists, 10 000 phenotype permutations, and gene-set sizes between 10 and 500. The plots were rendered with ggplot2 v3.5.0, heatmap v1.0.12, EnhancedVolcano v1.18.0 and UpSetR v1.4.0.

<u>scRNA-seq and analysis of small intestine lamina propria</u>

Leukocytes were isolated from small intestine lamina propria (as detailed above) and flow-sorted using a gating strategy to isolate $CD45^+$live cells. Cells were collected in low binding tubes containing PBS 0.05% (RNase-free BSA, Sigma, 126615-25ml). For each sample, an aliquot of cells was stained with acridine orange/propidium iodide Cell Viability Kit (Logos Biosystems, F23001) and counted with the LunaFx7 automatic cell counter (Logos Biosystems). Approximately [5000-30,000] cells were loaded on Chromium Chip and partitioned in nanolitre scale droplets using the Chromium X and Chromium GEM-X Single Cell Reagents (Chromium GEM-X Single Cell 3' v4 Gene Expression User Guide, CG000731). Within each droplet the cells were lysed, and the RNA was reverse transcribed. All the resulting cDNA within a droplet shared the same cell barcode. Illumina compatible libraries were generated from the cDNA using Chromium GEM-X Single Cell library reagents in accordance with the manufacturer's instructions (Chromium GEM-X Single Cell 3' v4 Gene Expression User Guide, CG000731). Final libraries are QC'd using the Agilent TapeStation and sequenced using the Illumina NovaSeq X. Sequencing read configuration: 28-10-10-90.

FASTQ files were aligned to the mm10 (GRCM38, version 93) transcriptome, and count matrices were generated, filtering for GEM cell barcodes (excluding GEMs with free-floating mRNA from lysed or dead cells) using Cell Ranger (version 9.0.0). All processing beyond this point was carried out using R version 4.4.1 using Seurat (version 5.2.1). Cells which did not meet the following criteria were removed; mitochondrial content within three standard deviations from the median, less than 500 genes detected per cell, and less than 1,000 RNA molecules detected per cell. Doublets were identified using DoubletFinder (version 2.0.4) and scDblFinder (version 1.20.2); cells called doublets by both methods were removed. Samples (4 biological replicates per Control and $C5^{\Delta Vav}$ genotypes) were integrated using the RunHarmony function, using 3000 variable genes and the pca reduction on the first 20 principal components; as determined using the intrinsicDimensions (version 1.2) package; to construct the UMAP. Clusters were identified using

a range of resolutions generating between 8 and 18 clusters. Clusters were annotated using a range of annotation packages at both the cluster and cell level: scCATCH (version 3.2.2); SingleR (version 2.8.0); clustifyr (version 1.18.0); scMCA (version 0.2.0) and cellTypist (version 1.6.3) and scanpy (version 1.10.4) within python (version 3.12.8). After inspection of marker genes and automated annotation results, a total of 12 clusters were identified using a resolution of 0.4. Marker genes per cluster were determined using the FindAllMarkers function, using a Wilcoxon rank-sum test, comparing each cluster to all other clusters. Differential gene expression between $C5^{\Delta Vav}$ mutants and Control samples per cluster was determined using the GlmGamPoi package, using aggregated expression after pseudobulking, considering the 4 replicates per genotype. Gene Set enrichment analysis using the differential genelist was done using the fgsea (version 1.32.2) package with pathway and biological processes genesets download from Broad Institute, ranking the genes using the log2FC. Genesets were deemed statistically significant if their adjusted p.value < 0.05. Cell-cell communication between clusters was determined using CellChat (version 2.1.2). CellChat was run individually for the Control and $C5^{\Delta Vav}$ samples, and major signalling changes across different Genotypes compared using quantitate contrasts and joint manifold learning, using the multiple datasets workflow. The cellchat analysis was performed as outlines in the cellchat vignette, with the population.size parameter set as 'TRUE', when computing the communication probability between clusters.

Ex vivo polyclonal activation of T cells

Dissociated mesenteric lymph nodes (mLNs) were isolated from $C5^{HetVav}$ or $C5^{\Delta Vav}$ were filtered through a 100µm cell strainer and counted with a Neubauer chamber. Lymphocytes were activated with CD3/CD28 coated beads following the manufacturer's instructions (Miltenyi Biotec-130-093-627). 10^6 mLN cells were incubated with 1:1 anti-CD3/CD28 coated beads for 24h at 37ºC, 5% CO_2 in 12 well plates (1mL total volume) with RPMI supplemeted with 10% fetal bovine serum (Gibco, 10270-106), L-glutamine (Stem Cell technologies, 07100), penicilin/streptramycin (Gibco, 15140-122), non-essential amino acids (Gibco, 11140-035), and sodium pyruvate (Gibco, 11360-039). After incubation, cells were stained with antibodies against CD3, CD4, CD8, CD69, CD44, and considered activated according to CD69 and CD44 expression in comparison to inactivated controls.

Lymphocyte transfer model of intestinal inflammation

Splenic lymphocytes were obtained from heterozygotes or homozygotes *Arpc5*$^{Cre\text{-}Foxp3\text{-}YFP}$ mice and $CD45^{rbhi}$ or Foxp3-YFP positive cells were FACS sorted and transferred into $Rag^{-/-}$ mice. Positive control mice only received $5x10^5$ $CD45^{rbhi}$ cells while adoptive transfer of $2x10^5$ Foxp3-YFP cells were given in the other groups as indicated in the figure. Mice weight was monitored and humane endpoint observed when reaching a 20% loss from starting weight.

Differentiation of bone marrow derived macrophages

Bone marrow was collected from the tibia and femur of male and female mice at 6 – 10 weeks and differentiated in vitro for 7 days in RPMI (Gibco R8758) supplemeted with 10% fetal bovine serum (Gibco, 10270-106), 10% L929 conditioned medium (Crick Cell services STP), L-glutamine (Stem Cell technologies, 07100), penicilin/streptramycin (Gibco, 15140-122), non-essential amino acids (Gibco, 11140-035), and sodium pyruvate (Gibco, 11360-039) to obtain bone marrow derived macrophages (BMDM).

Phagocytosis, bacterial killing and cytotoxicity assays in BMDM

To assess bacterial phagocytosis, adhered BMDMs (5 x 10^5) were incubated with FITC-Heat-killed (HK) *E.coli* (60μg/mL-E2861-Invitrogen) or pHrodo *E.coli* (1μg/mL-P35366-Invitrogen) for up to 120 min at 37ºC, 5% CO_2. Negative controls were incubated at 4ºC during the same period. Bacterial uptake was assessed by flow cytometry using the phagocytosis index calculation as follows: [(MFI x % positive cells at 37ºC) – (MFI x % positive cells at 4ºC)].

For bacterial killing (3x10^5 BMDMs in 12 well plates) and cytotoxicity assays (10^5 BMDMs in 96 well plate) adherent BMDM were incubated with phase *E. coli* K-12 (NCTC_10538) mid-log at MOI 5, 10 or 50. The bacteria concentration was assessed using OD_{600}. The BMDM killing capacity was assessed by microscopy. Ten random fields of view were imaged on a Zeiss Axio Observer spinning-disk microscope equipped with a Plan Achromat 63×/1.4 Ph3 M27 oil lens, an Evolve 512 camera, and a Yokagawa CSUX spinning disk. The microscope was controlled by the SlideBook software (3i Intelligent Imaging Innovations). Images were analyzed using FIJI and internalized bacteria were detected using anti-E coli (abcam, ab137967). Microscopy secondary antibodies (A31572, Thermo Scientific) and DAPI (4083S, Cell Signalling Technology). Calculations were obtained as follows: [(internalized bacteria at T0) – (internalized bacteria at Time X)].

The BMDMs cytotoxicity was assessed using the CyQUANT^{Tm}LDH Cytotoxicity assay following the manufacturer's instructions (Thermo Fisher Scientific, C20300). For G-actin or F-actin detection, BMDMs (5x10^5 in non-adherent 6 well plates) were incubated with E. coli K-12 (MOI 50) for 15 min or vehicle. 30 min pre-treatment with 10μM Cytochalasin D (Merck, 250255) was performed when indicated. After treatment cells were permeabilized using the Foxp3/Transcription Factor Staining Buffer Set (00-5523-00 ThermoFisher Scientific) and stained with antiDNAse-I Alexa Fluor 488 (Invitrogen, D12371) or Alexa Fluor 568-phalloidin (Molecular Probes), respectively.

Morphological and live cell imaging of BMDM

BMDM$^{LifeAct\text{-}GFP}$ derived from C5^{HetVav} or C5$^{\Delta Vav}$ murine bone marrow were seeded on fibronectin coated 35 mm Matek dish at 5x10^4 cells/well and incubated overnight at 37°C with 5% CO_2. Cells were imaged as described above. Data for 10-12 cells per sample were segmented using ilastik and segmented images were then analyzed using FIJI. For live cell imaging the cells were seeded on fibronectin coated ibidi 4-well glass-bottom μ-slide at 4x10^4 cells/well and incubated overnight at 37°C with 5% CO_2. pHrodo™ Deep Red E. coli BioParticles™ Conjugate (Thermo Fisher) was prepared according to manufacturer's instructions. Prior to imaging, 5 μL of pHrodo™ Deep Red E. coli was added into each well. The imaging chamber was maintained at 37°C. Both 2D and 3D live cell imaging of phagocytosis used a Zeiss Axio Observe microscope, an Evolve 512 camera and a Yokagawa CSUX spinning disk. For 2D live cell imaging, cells were imaged at a single z plane every 500 ms for 3 minutes using a Plan Achromat 100x/1.46 lens. For 3D live cell imaging, cells were images every 10 s for 4 minutes, with Z-stack of 0.27 μm steps over 10 μm with a Plan Achromat5 63x/1.4 Ph3 M27 oil lens. The microscope was controlled by the SlideBook software (3i Intelligent Imaging Innovations). The 2D videos were generated using Fiji and the 3D videos were reconstructed using napari.

Statistical analysis

Data are shown as the mean ± S.E.M. Sample sizes were established prior to experimental approach with help of the experimental design assistant of NC3Rs to achieve statistical power while minimizing animal use. To test normality or lognormality we applied D'Agostino & Pearson test. All statistical comparisons were performed using Prism 10 (GraphPad) with specific statistical test applied as stated in the figure legends. Statistical significance was considered as $P<0.05$.

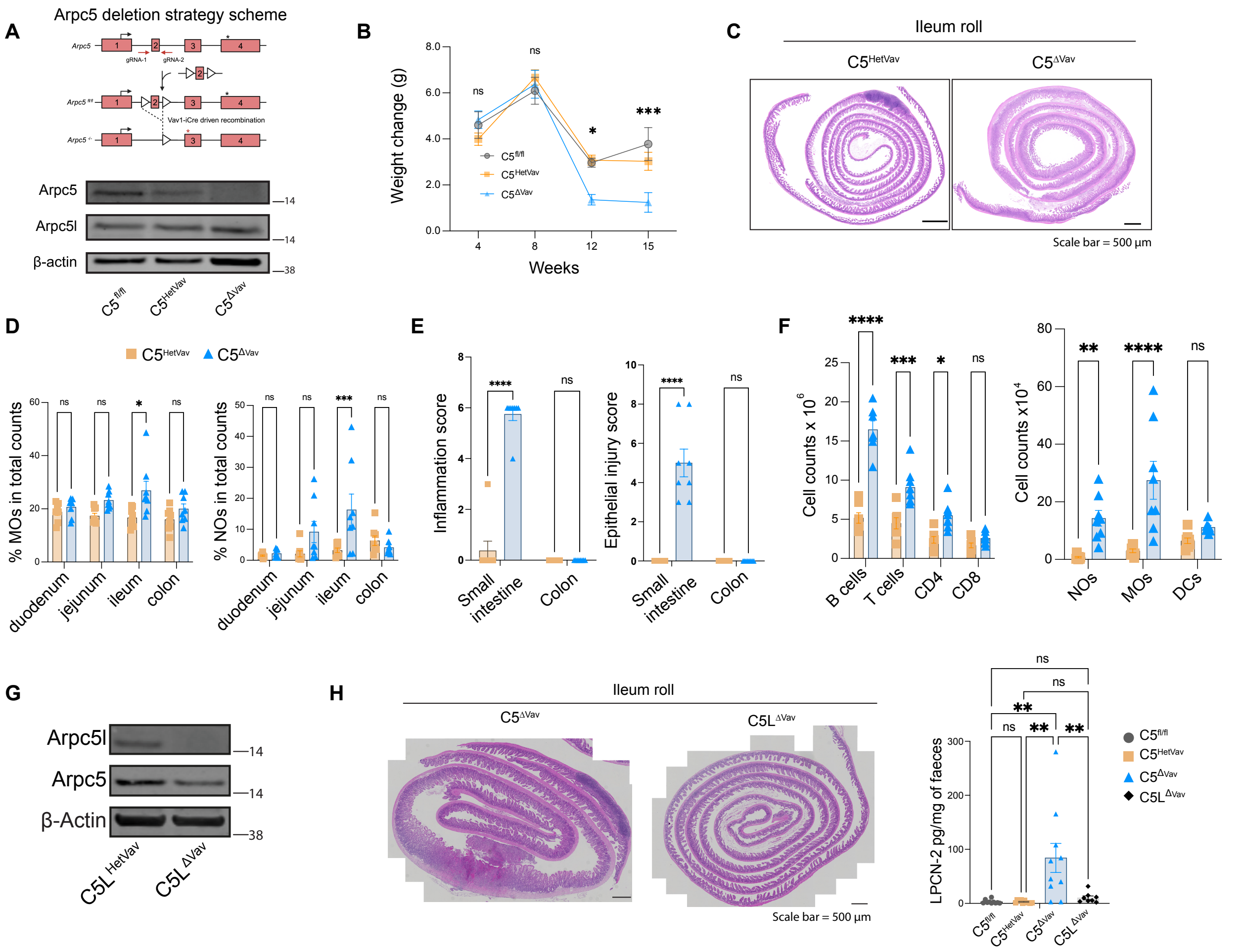

Fig. S1. (**A**) Arpc5 deletion strategy using Vav1-iCre and the level of Arpc5 and Arpc5l in $C5^{fl/fl}$, $C5^{HetVav}$ or $C5^{\Delta Vav}$ bone marrow extracts. (**B**) Weight change (g) of $C5^{fl/fl}$, $C5^{HetVav}$ or $C5^{\Delta Vav}$ mice over 15 weeks. (**C**) Representative H&E images of the ileum rolls. (**D**) Macrophages and neutrophils quantification in different intestinal sections (**E**) Quantification of the histopathology score of intestines of indicated 8-15 wks old mice. (**F**) Cell numbers of mesenteric lymph nodes (mLNs) B and T cells, $CD4^{+}$ and $CD8^{+}$, macrophages (MOs), neutrophils (NOs) and dendritic cells (DCs) in the indicated mice. (**G**) The levels of Arpc5 and Arpc5l in $C5L^{HetVav}$ or $C5L^{\Delta Vav}$ bone marrow extracts, actin is a loading control. (**H**) Representative H&E images of ileum rolls and Lipocalin-2 (LPCN-2) faecal levels in the indicated mice (including values also shown in Fig.1A, $C5L^{\Delta Vav}$ n=8). Representative gating strategies used in this figure are shown in Figures S10-12. Full scans of the original immunoblots are shown in Figure S13. Animals assessed in B were 4–15-week-old mice, n=12 of at least three experiments and datapoints represent the variance of weight change. For all the other panels, data shown of at least three independent experiments, n=8. In graphs, each data point represents data from an individual mouse, bars show the mean and error bars ± SEM. Statistical analysis was performed using two-way analysis of variance (ANOVA) in B; Multiple Mann-Whitney test in D, E, F or one-way ANOVA in H. Significant p values are indicated on the graphs. C5fl/fl=Arpc5fl/fl, $C5^{HetVav}$=$Arpc5^{HetVav}$, C5ΔVav=$Arpc5^{\Delta Vav}$ and $C5L^{\Delta Vav}$=$Arpc5l^{\Delta Vav}$. Scale bars = 500µm. ns = not significant. * = p-value < 0.05. ** = p-value < 0.01. *** = p-value < 0.001 and **** = p-value < 0.0001.

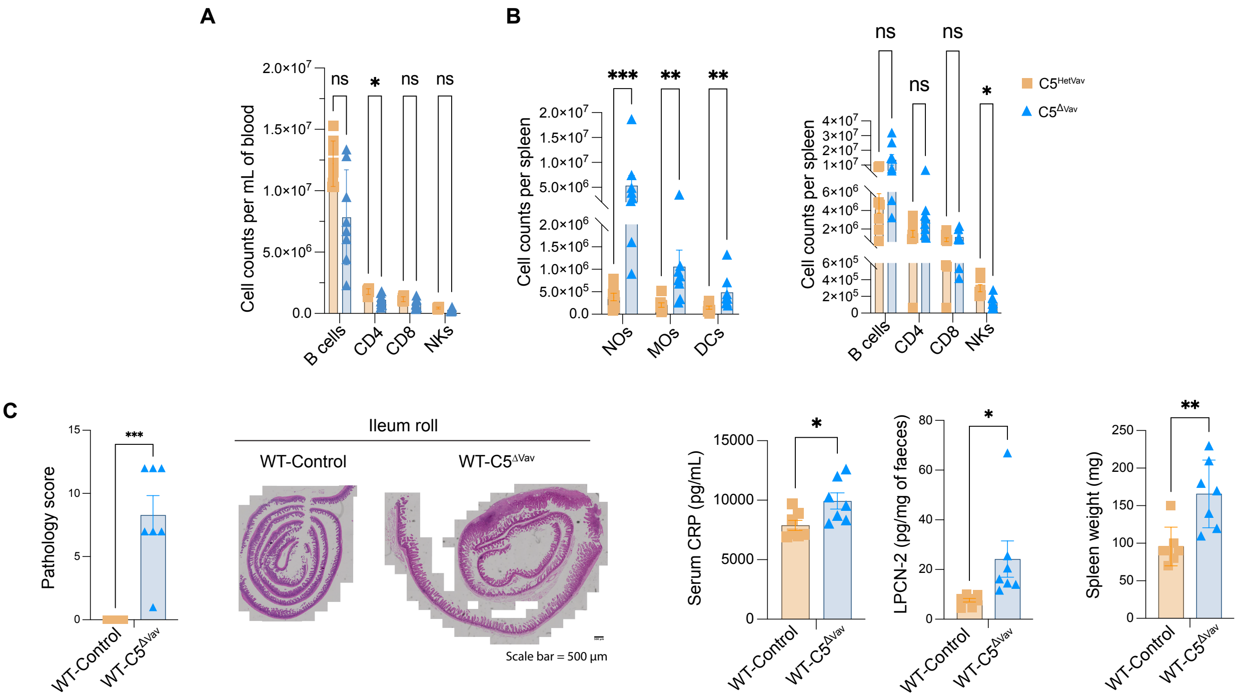

Fig. S2. (**A**) Cell numbers of B, CD4$^+$, CD8$^+$ and natural killer (NK) lymphocytes in the blood of indicated mice. (**B**). NOs, MOs, DCs, B lymphocytes, CD4$^+$, CD8$^+$ and NK cells numbers in the spleen of C5^{HetVav} or C5$^{\Delta Vav}$ mice. (**C**) Representative H&E images and pathological score of ileum rolls of indicated animals after bone marrow transplantation. Quantification of the levels of C-reactive protein (CRP) in the serum, LPCN-2 in the faeces and spleen weight in indicated animals after bone marrow transplantation. Representative gating strategies used in this figure are shown in Figure S10-12. In graphs, data shown of at least three independent experiments, n=7. Each data point represents data from an individual mouse, bars show the mean and error bars ± SEM. Statistical analysis was performed using Multiple Mann-Whitney test in A and B; Mann-Whitney test in C and significant p values are indicated on the graphs. C5$^{fl/fl}$=Arpc5$^{fl/fl}$), C5^{HetVav}=Arpc5^{HetVav}), C5$^{\Delta Vav}$=Arpc5$^{\Delta Vav}$ and C5L$^{\Delta Vav}$=Arpc5l$^{\Delta Vav}$. Scale bars = 500μm. ns = not significant. * = p-value < 0.05. ** = p-value < 0.01. *** = p-value < 0.001 and **** = p-value < 0.0001.

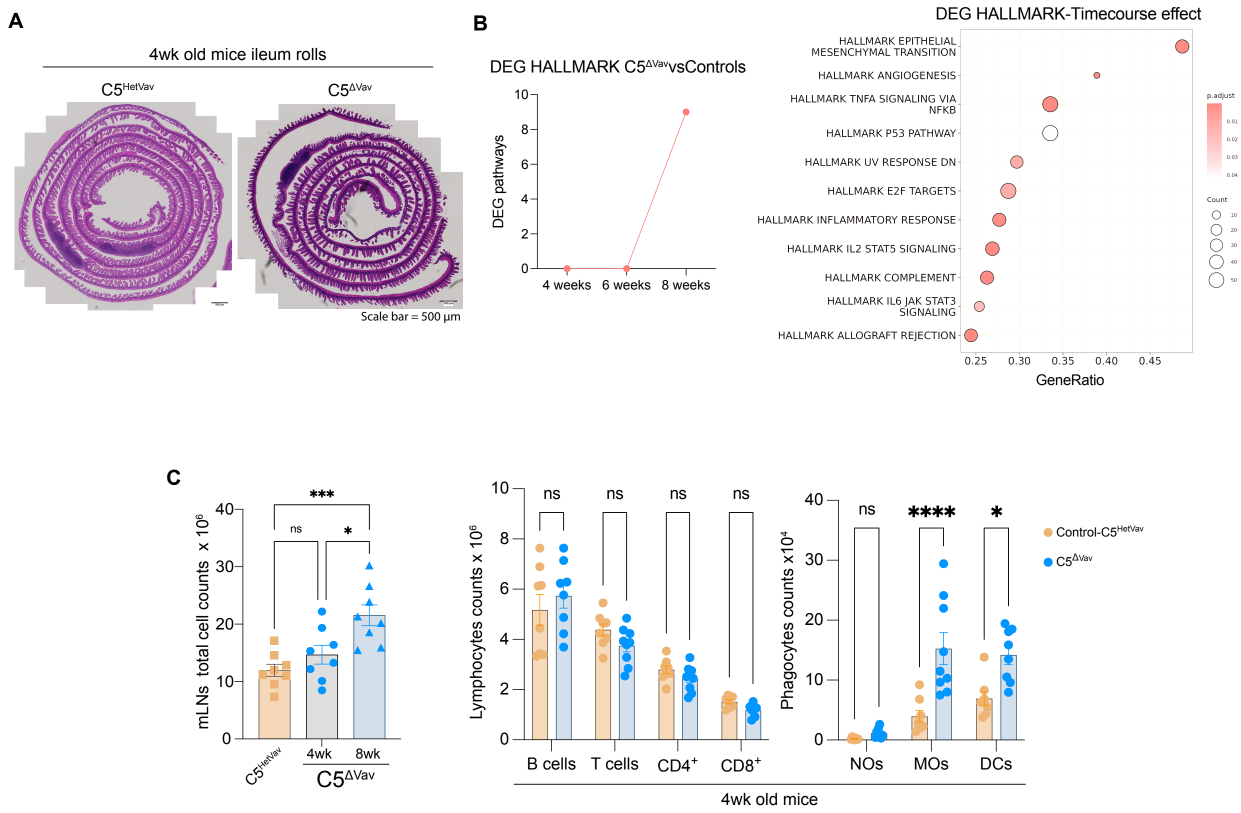

Fig. S3. (**A**) Representative H&E image of ileum rolls of 4-week-old (wk) C5^{HetVav} or C5$^{\Delta Vav}$ mice. (**B**) Number of differentially expressed gene (DEG) pathways (left) and enriched DEG hallmark (right) observed in intestinal lamina propria macrophages obtained from C5^{HetVav} or C5$^{\Delta Vav}$ mice over the time. (**C**) Total cell numbers in mLNs of 4- and 8-week-old (left) together with the cell counts of B, T and CD4^{+} or CD8^{+} lymphocytes (middle), neutrophils (NOs), macrophages (MOs) or dendritic cells (DCs) (right) in mLNs from 4 week (wk) old C5^{HetVav} or C5$^{\Delta Vav}$ mice. Representative gating strategy used in this figure is shown in Figure S12. In graphs, data shown of at least three independent experiments, n=8. Each data point represents data from an individual mouse, bars show the mean and error bars ± SEM. Statistical analysis was performed using Kruskal-Wallis test in C left; Multiple Mann-Whitney test was performed in C right. Significant p values are indicated on the graphs. Controls used were C5^{HetVav} (Arpc5^{HetVav}). C5$^{\Delta Vav}$=Arpc5 $^{\Delta Vav}$, Veh-C5$^{\Delta Vav}$=Arpc5$^{\Delta Vav}$ treated with vehicle, and Atbx-C5$^{\Delta Vav}$=Arpc5$^{\Delta Vav}$ treated with antibiotics. Scale bars = 500µm. ns = not significant. * = p-value < 0.05. ** = p-value < 0.01. *** = p-value < 0.001 and **** = p-value < 0.0001.

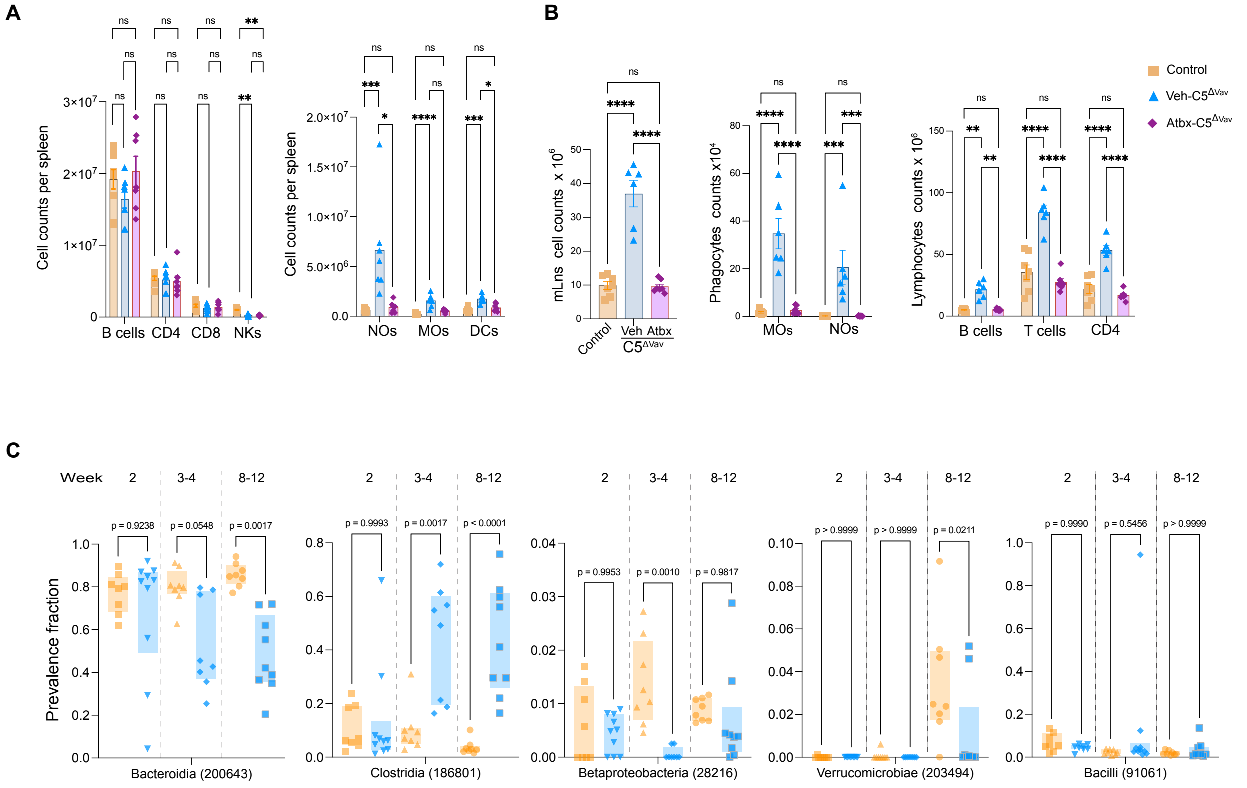

Fig. S4. (**A**) Splenic B, T and natural killer (NK) lymphocytes (left), NOs, MOs and DCs (right) cell counts in the indicated mice after antibiotic treatment. (**B**) mLNs total cell counts (left), together with NOs and MOs (middle), B and T lymphocytes (right) cell numbers from mLNs of mice treated with antibiotic. (**C**) Prevalence fraction of most abundant microbial classes found in indicated mice intestines. Representative gating strategies used in this figure are shown in Figure S10-12. In graphs, data shown of at least three independent experiments, n=7 for A-B and n=8 for C. Each data point represents data from an individual mouse, bars show the mean and error bars ± SEM. Statistical analysis was performed using Kruskal-Wallis test in A and B. One-way ANOVA test was performed in C. Significant p values are indicated on the graphs. Controls used were $C5^{HetVav}$ ($Arpc5^{HetVav}$). $C5^{\Delta Vav}$ =$Arpc5^{\Delta Vav}$, Veh-$C5^{\Delta Vav}$ =$Arpc5^{\Delta Vav}$ treated with vehicle, and Atbx-$C5^{\Delta Vav}$ =$Arpc5^{\Delta Vav}$ treated with antibiotics. Scale bars = 500µm. ns = not significant. * = p-value < 0.05. ** = p-value < 0.01. *** = p-value < 0.001 and **** = p-value < 0.0001.

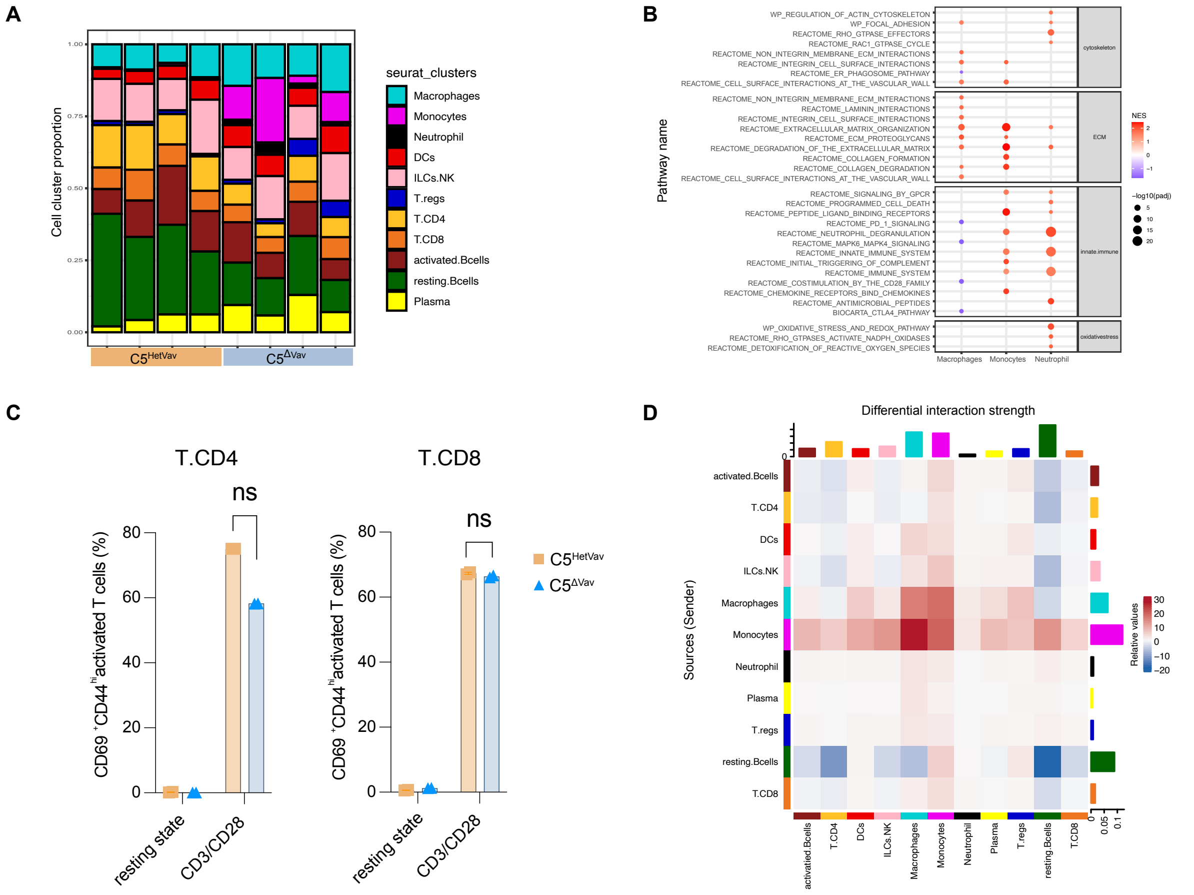

Fig. S5. (**A**) Bar plots representing the proportion of immune cells per biological replicate in $C5^{HetVav}$ or $C5^{\Delta Vav}$ mice. (**B**) Biological relevant pathways of differentially expressed genes in Macrophages, Monocytes and Neutrophils from $C5^{HetVav}$ ($Arpc5^{HetVav}$) and $C5^{\Delta Vav}$ ($Arpc5^{\Delta Vav}$) animals. (**C**) Anti CD3/CD28 polyclonal activation assay in T cells from $C5^{HetVav}$ and $C5^{\Delta Vav}$ animals. (**D**) Representation of the differential interaction strength obtained from CellChat analysis. In A, B and D data representative from one experiment, n=4 (41130 cells per group). In C every datapoint represents data from two independent experiments shown as means. Mann-Whitney test was performed in C. ns = not significant.

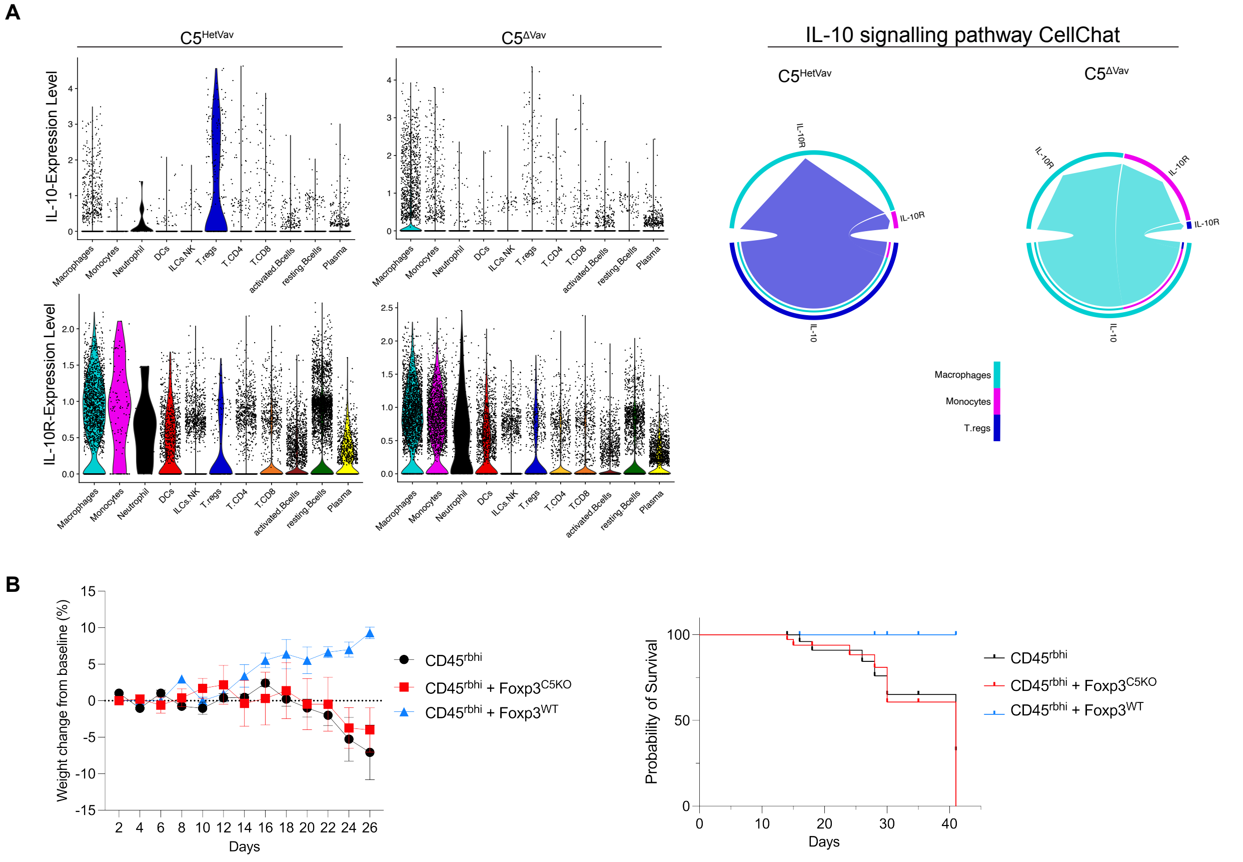

Fig. S6. (**A**) Expression levels of interleukin-10 (IL-10) and IL-10 receptor (IL-10R) (left). IL-10 signalling communication with outgoing (IL-10 production) and incoming (IL-10 recognition) interactions between T regulatory cells (Tregs), Macrophages and Monocytes inferred using CellChat (right). (**B**) Tregs function in the lymphocyte transfer model of intestinal inflammation. $CD45^{rbhi}$, $CD45^{rbhi}$ + $Foxp3^{WT}$ or $CD45^{rbhi}$ + $Foxp3^{C5KO}$ were transplanted into $Rag^{-/-}$ mice and weight change and survival probability assessed over four weeks. In A data shown from one experiment, n=4. In B every datapoint represent means ± SEM of three independent experiments. For $CD45^{rbhi}$ and $CD45^{rbhi}$ + $Foxp3^{C5KO}$ n=8. For $CD45^{rbhi}$ + $Foxp3^{WT}$ n=6. ns = not significant.

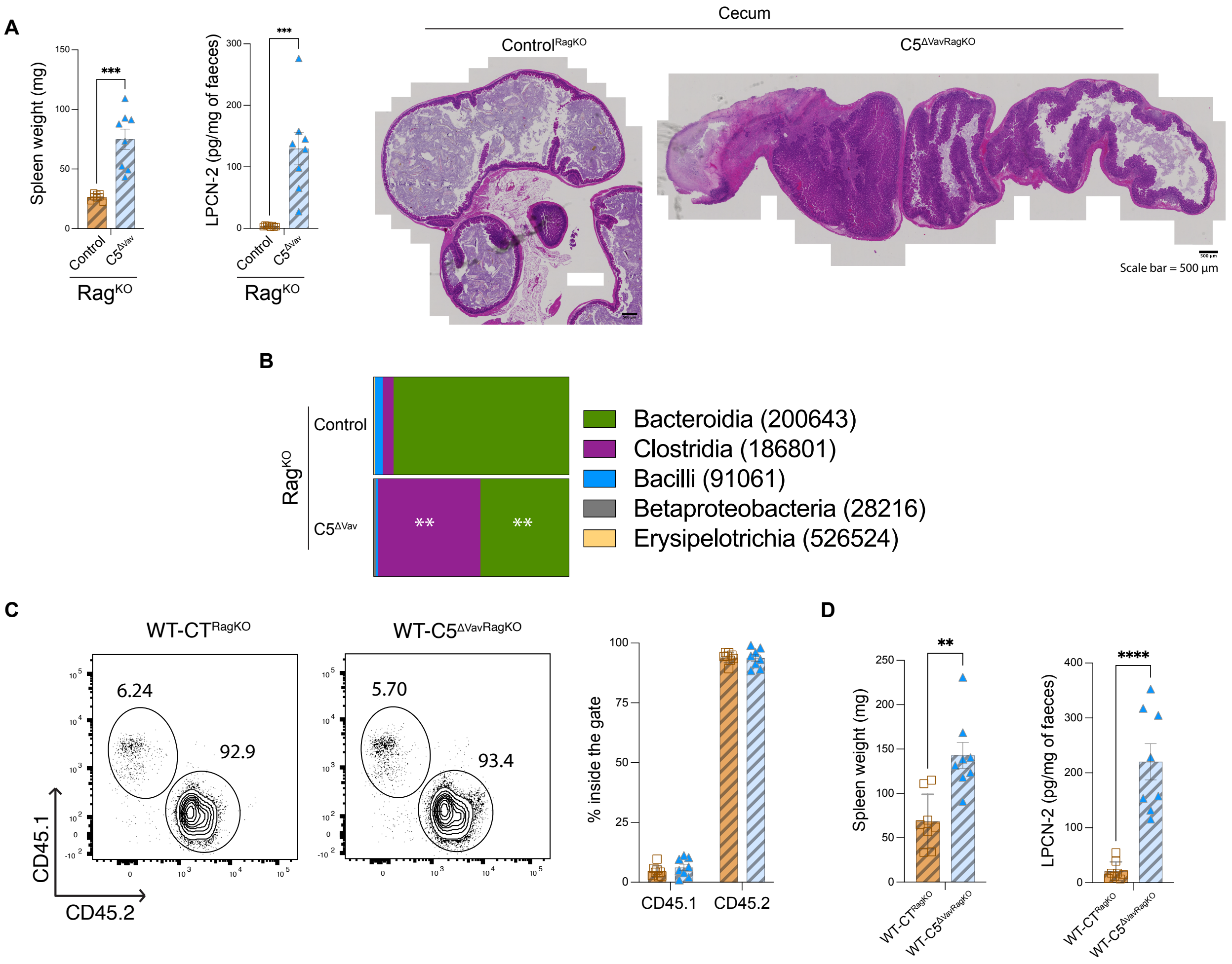

Fig. S7. (**A**) Spleen weight, faecal lipocalin-2 (LPCN-2) levels (left) and representative H&E images of cecum of control^{RagKO} or C5$^{\Delta VavRagKO}$ mice (right). (**B**) Most abundant classes of bacteria found in the intestinal microbiome analysis of control^{RagKO} or C5$^{\Delta VavRagKO}$. (**C**) Representative Facs plots (left) and quantification of CD45.1^{+} or CD45.2^{+} of mice after bone marrow transplantation (right). (**D**) Spleen weight (left) and faecal lipocalin-2 (LPCN-2) levels (right) in indicated mice after bone marrow transplantation. Each datapoint represent individual animal. Data shown as means ± SEM of at least three independent experiments, n=8, except in B n=5 from two independent experiments. Statistical analysis was performed using Mann-Whitney test in A, B, D. Significant p values are indicated on the graphs. Controls used were Arpc5^{HetVav}, C5$^{\Delta Vav}$ (Arpc5$^{\Delta Vav}$). Scale bars are indicated in the figure. ns = not significant. * = p-value < 0.05. ** = p-value < 0.01. *** = p-value < 0.001 and **** = p-value < 0.0001.

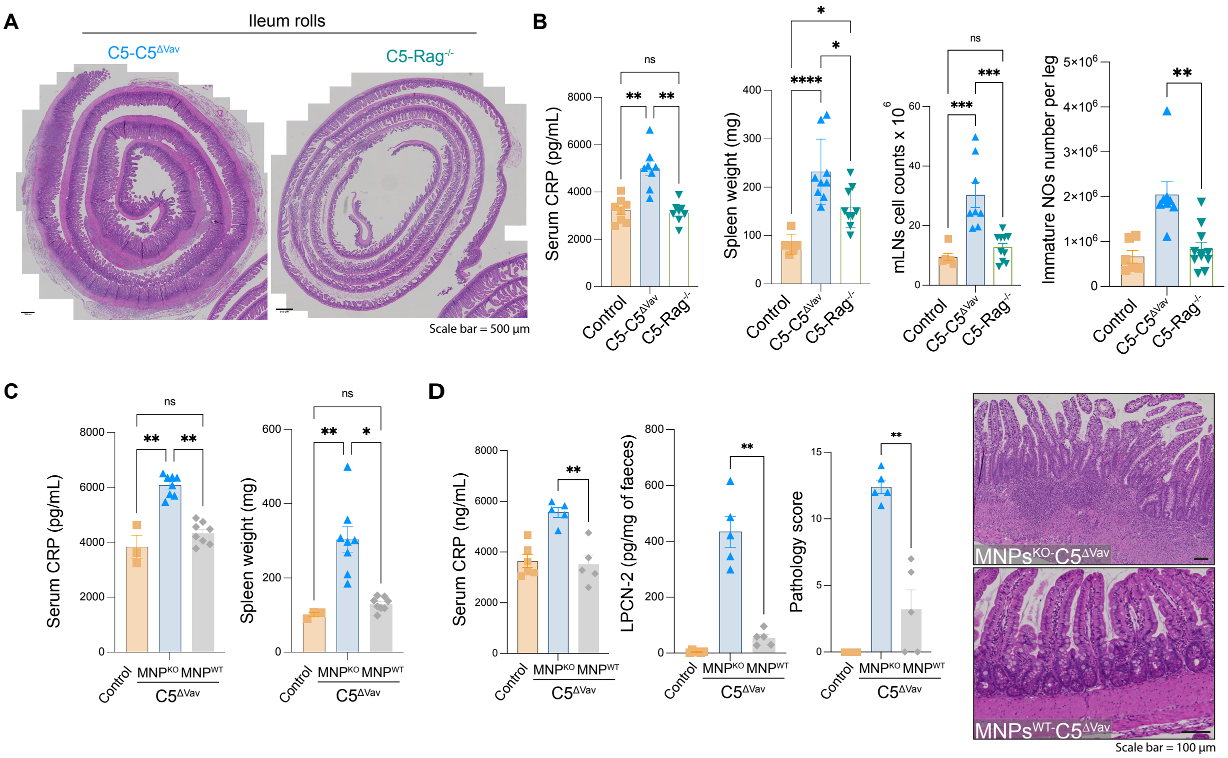

Fig. S8. (**A**) Representative image of H&E images of ileum rolls. (**B**) Quantification of the levels of serum C-reactive protein (CRP), spleen weight, mLNs cell counts and immature neutrophils (NOs) counts in the bone marrow of indicated animals after busulfan adoptive transfer. (**C**) CRP levels in the serum and spleen weight of indicated animals after busulfan depletion followed by mononuclear phagocytes (MNPs) adoptive cell transfer (Control n=3 and other groups n=8). (**D**) CRP levels in the serum, LPCN-2 faecal levels and histopathology score together with representative H&E images of the ileum of clodronate liposomes depleted 4-week-old animals followed by wild-type (MNP^{WT}) or $C5^{\Delta Vav}$ (MNP^{KO}) transplantation. Every datapoint represent individual animal. Data shown as means ± SEM of at least three independent experiments, n=6-11, except in D where n=5 from two independent experiments. Statistical analysis was performed using Mann-Whitney in B (right) and in D; Kruskal-Wallis test in B and C. Significant p values are indicated on the graphs. Controls used were $Arpc5^{HetVav}$, $C5^{\Delta Vav}$ ($Arpc5^{\Delta Vav}$). Scale bars are indicated in the figure. ns = not significant. * = p-value < 0.05. ** = p-value < 0.01. *** = p-value < 0.001 and **** = p-value < 0.0001.

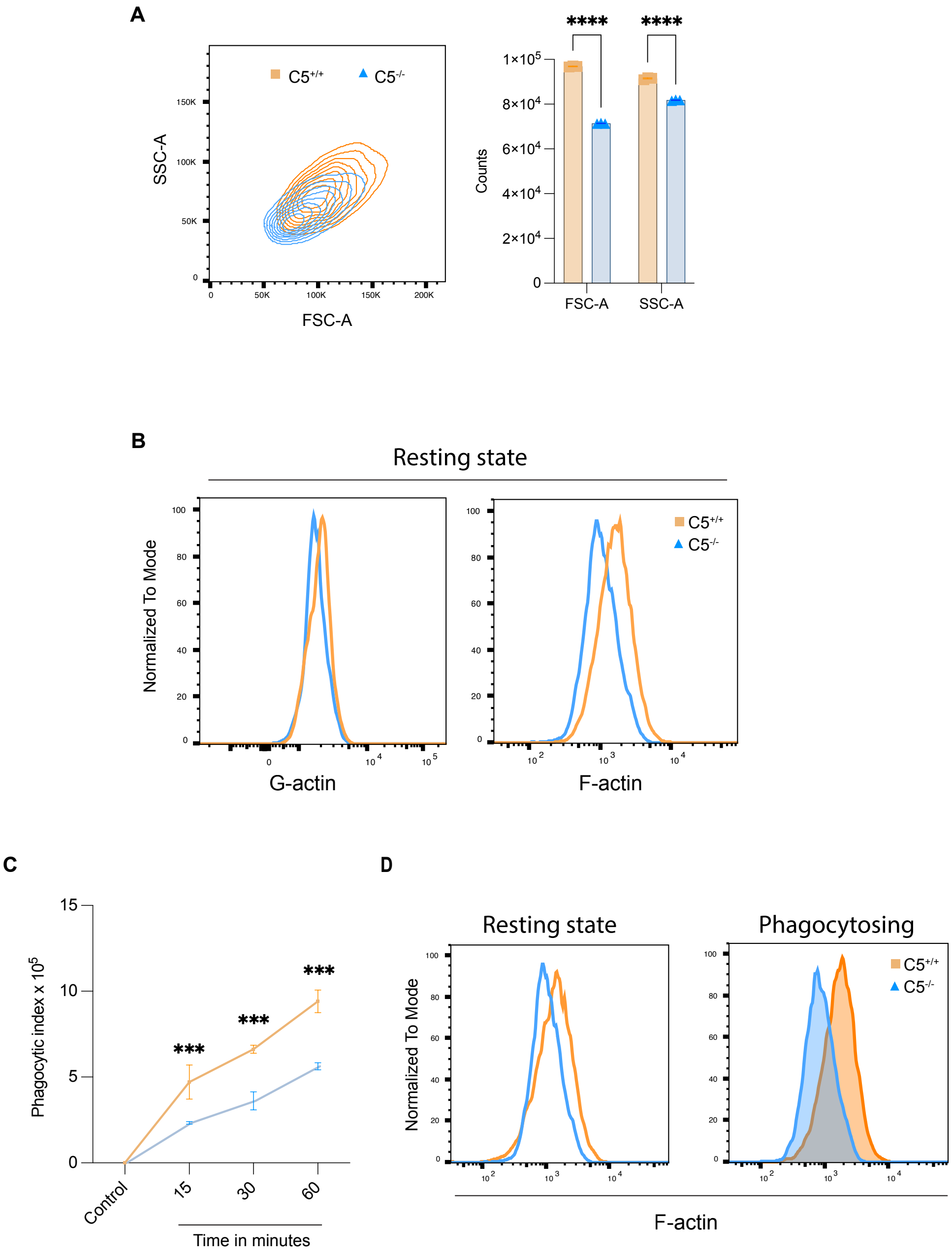

Fig. S9. (**A**) Representative Facs plot and measurements of side scatter area (SSC-A) or forward scatter area (FSC-A) of indicated bone marrow derived macrophages (BMDMs). (**B**) Facs histograms of G-actin or F-actin in unstimulated BMDMs. (**C**) Quantification of *E coli*-FITC uptake by $C5^{+/-}$ or $C5^{-/-}$ BMDMs in the indicated timepoints. (**D**) Representative F-actin Facs histograms of *E.coli*-activated BMDMs. Data shown as means ± SEM of a representative of at least three independent experiments. Statistical analysis was performed using Mann-Whitney test in A and two-way ANOVA in C. Significant p values are indicated on the graphs. $C5^{+/-}$ = $Arpc5^{+/-}$, $C5^{-/-}$ = $Arpc5^{-/-}$. *** = p-value < 0.001 and **** = p-value < 0.0001.

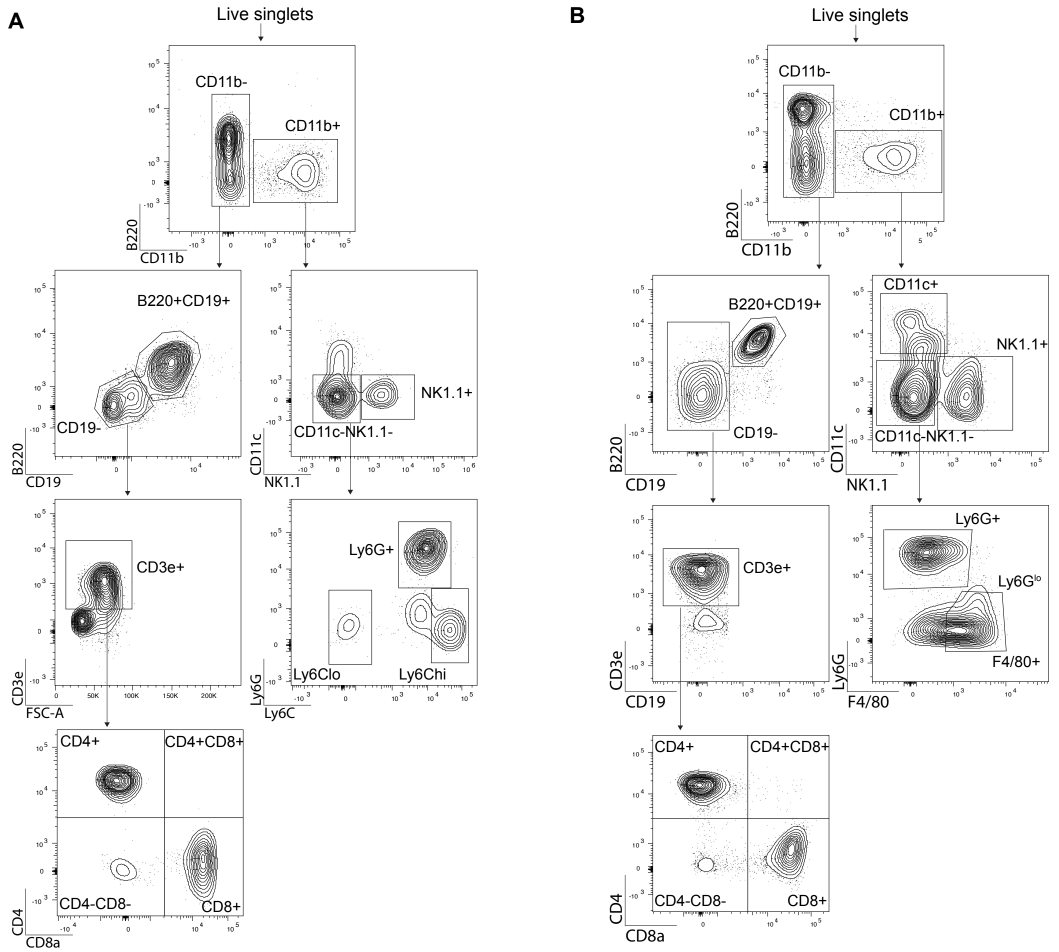

Figure S10. (**A**) Representative flow cytometry plots showing the gating strategy used to quantify leukocytes in peripheral blood shown in Fig.1G and Fig.S1. All samples were initially gated based on forward scatter, side scatter and live-dead dye fluorescence to identify live single cells. Neutrophils (NOs) (B220$^-$CD11b$^+$CD11c$^-$NK1.1$^-$Ly6G$^{hi/lo}$), monocytes (MOs) (B220$^-$CD11b$^+$CD11c$^-$NK1.1$^-$Ly6G$^-$Ly6C$^{hi/lo}$), B cells (B220$^+$CD19$^+$), CD4$^+$ T cells (B220$^-$CD19$^-$CD3e$^+$CD4$^+$CD8a$^-$), CD8$^+$ T cells (B220$^-$CD19$^-$CD3e$^+$CD4$^-$CD8a$^+$) and natural killer cells (NKs) (B220$^-$CD11b$^+$CD11c$^-$NK1.1$^+$) were gated. (**B**) Representative flow cytometry plots showing the gating strategy used to quantify leukocytes in spleen shown in Fig. S1J and S2D. All samples were initially gated based on forward scatter, side scatter and live-dead dye fluorescence to identify live single cells. Neutrophils (NOs) (B220$^-$CD11b$^+$CD11c$^-$NK1.1$^-$Ly6G$^{hi/lo}$), macrophages (MOs) (B220$^-$CD11b$^+$CD11c-NK1.1-Ly6G-F4/80+), dendritic cells (DCs) (B220-CD11b$^+$ NK1.1$^-$CD11c$^+$), B cells (B220$^+$CD19$^+$), CD4$^+$ T cells (B220$^-$CD19$^-$CD3e$^+$CD4$^+$CD8a$^-$), CD8$^+$ T cells (B220$^-$CD19$^-$CD3e$^+$CD4$^-$CD8a$^+$) and natural killer cells (NKs) (B220$^-$CD11b$^+$CD11c$^-$NK1.1$^+$) were gated.

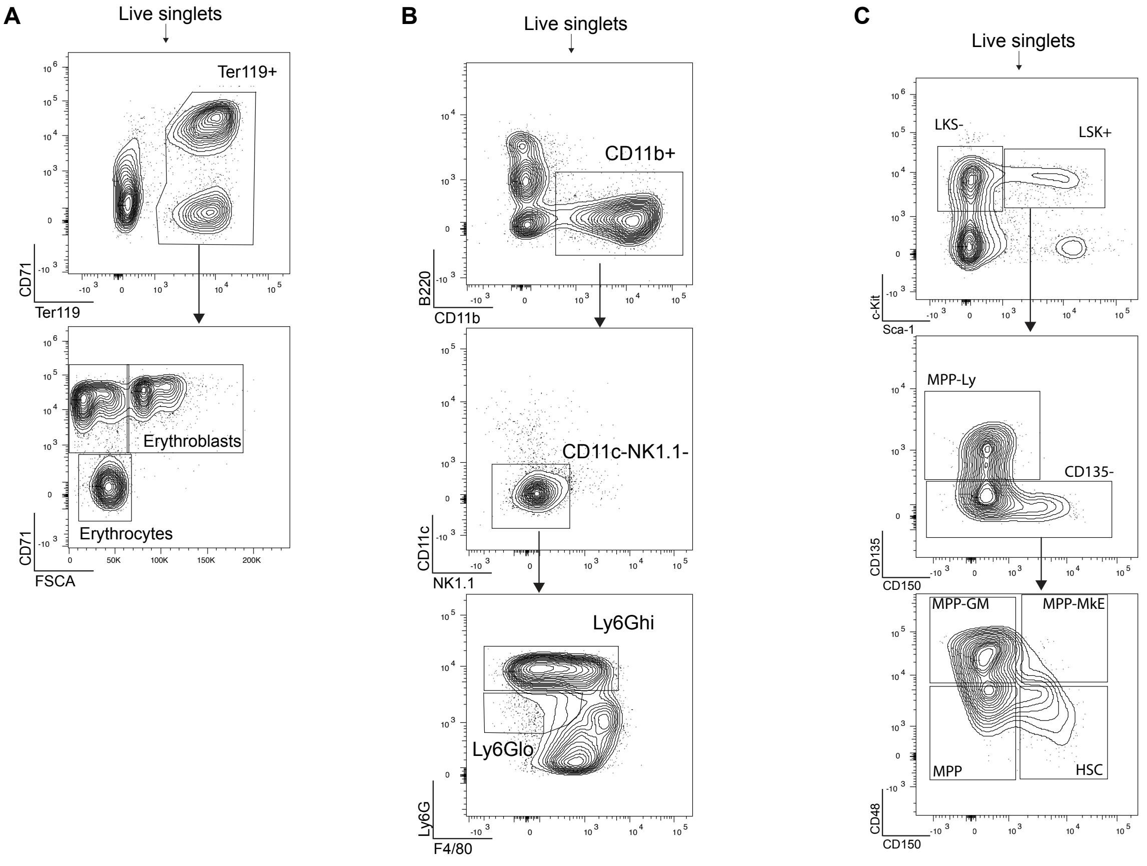

Fig. S11. (**A**) Representative flow cytometry plots showing the gating strategy used to quantify erythroid lineage cells in bone marrow shown in Fig.1H (middle). All samples were initially gated based on forward scatter, side scatter and live-dead dye fluorescence to identify live single cells. CD71, Ter119 and forward scatter were used as markers to identify erythroid lineage cells. Ter119^{hi}CD71^{hi} the erythroblasts; Ter119^{hi}CD71^{lo}gate denotes the erythrocytes. (**B**) Representative flow cytometry plots showing the gating strategy used to quantify neutrophils in bone marrow shown in Fig.1H (right), 2G (right) and Fig.S8B (right). All samples were initially gated based on forward scatter, side scatter and live-dead dye fluorescence to identify live single cells. CD11b^{+}B220^{-}CD11c^{-}NK1.1^{-}Ly6G^{lo} gate denotes the immature neutrophils; CD11b^{+}B220^{-}CD11c^{-}NK1.1^{-}Ly6G^{hi} gate denotes the mature neutrophils. (**C**) Representative flow cytometry plots showing the gating strategy used to quantify HSPCs including hematopoietic stem cells (HSCs) and multipotent progenitors (MPPs) shown in Fig.1I. All samples were initially gated based on forward scatter, side scatter, live-dead dye fluorescence and lineage (lin) cocktails (B220, CD2, Gr-1, Ter119) to identify single live lineage negative cells. Gating strategy to identify HSPC was adopted as follows: lin^{-}c-Kit^{+}Sca-1^{+} gate denotes the LSK cells. From the LSK population, CD135^{+}CD150^{-} denotes the lymphoid-biased MPPs (MPP-Ly); CD135-CD48^{+}CD150^{-} denotes the myeloid-biased MPPs (MPP-GM); CD135^{-}CD48^{+}CD150^{+} denotes the megakaryocyte and erythroid-biased-MPPs (MPP-MkE); CD135^{-}CD48^{-}CD150^{-} denotes the unbiased MPPs (MPP); and CD135^{-}CD48^{-}CD150^{+} denotes the HSCs.

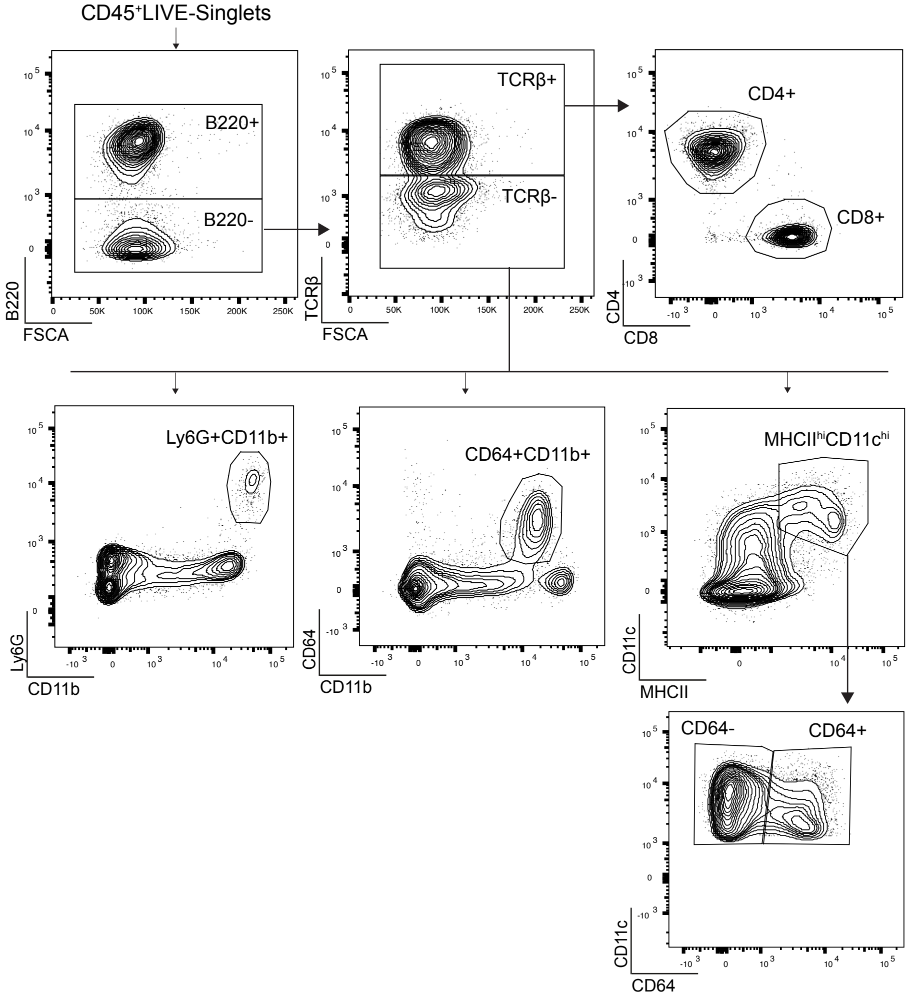

Fig. S12. Representative flow cytometry plots showing the gating strategy used to quantify leukocytes in mesenteric lymph nodes (mLNs) shown in Fig. S1F, S2C and S2E. All samples were initially gated based on forward scatter, side scatter, live-dead fluorescence dye to identify live CD45^{+} single cells. B cells (B220^{+}), T cells (B220^{-}TCRβ^{+}), CD4^{+}T cells (B220^{-}TCRβ^{+}CD4^{+}CD8a^{-}), CD8^{+}T cells (B220^{-}TCRβ^{+}CD4^{-}CD8a^{+}), Neutrophils (NOs) (B220^{-}TCRβ^{-}CD11b^{+}Ly6G$^{hi/lo}$), macrophages (MOs) (B220^{-}TCRβ^{-}CD11b^{+}CD64^{+}), dendritic cells (DCs) (B220^{-}TCRβ^{-}CD64^{-}CD11c^{hi}MHCII^{hi}) were gated.

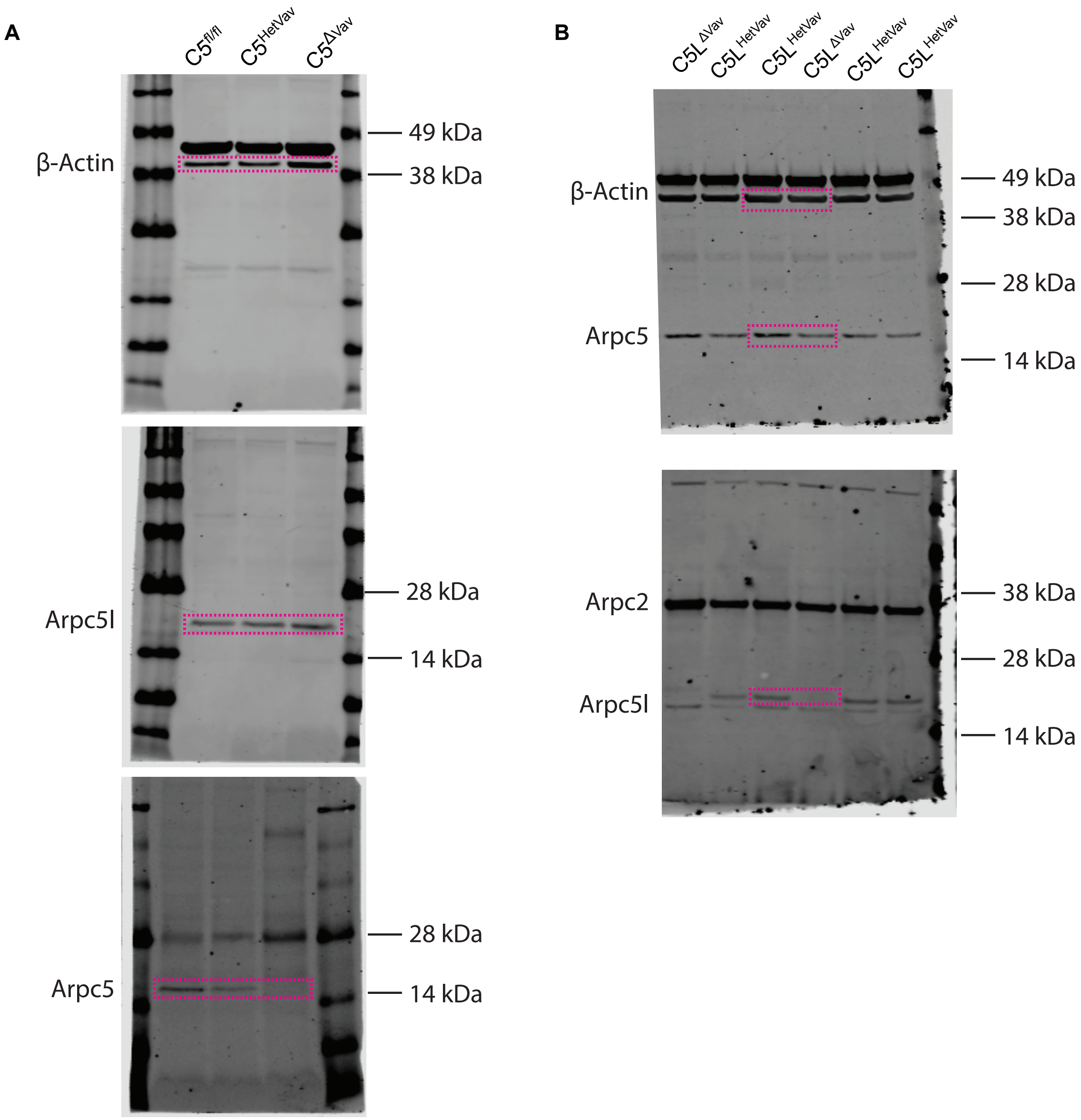

Figure S13. (**A**) Full scans of the original immunoblots used for the data presented in Fig.S1A. (**B**) Full scans of the original immunoblots used for the data presented in Fig.S1G. Magenta boxes indicate the cut regions shown in Fig.S1G. Molecular weight markers (kDa) are indicated. Blots were processed and imaged under the same conditions across samples unless otherwise indicated.

Movie S1. Phagocytosis of E.coli by BMDMs with and without Arpc5. Representative movie showing actin dynamics in $Arpc5^{+/-}$ (left) and $Arpc5^{-/-}$ (right) BMDMs expressing GFP-LifeAct (Cyan) during phagocytosis of *E. coli* (Magenta). White arrows indicate the bacteria being phagocytosed. The time in minutes and seconds is shown on the upper left corner. Scale bar = 10 µm. Image stills from the movie are shown in Fig.4D.

Movie S2. 3D view of *E. coli* uptake by BMDMs. Representative movie showing *E. coli* (Magenta) uptake in BMDMs expressing GFP-LifeAct (Cyan) in $Arpc5^{+/-}$ (left) and $Arpc5^{-/-}$ (right) BMDM. The time in minutes is shown on the upper left corner and the scale bar is shown on the lower right corner. Image stills from the movie are shown in Fig.4E.

References and Notes

1. E. G. G. Sprenkeler, S. D. S. Webbers, T. W. Kuijpers, When actin is not actin' like it should: A new category of distinct primary immunodeficiency disorders. *J. Innate Immun.* **13**, 3–25 (2021).

2. E. Janssen, R. S. Geha, Primary immunodeficiencies caused by mutations in actin regulatory proteins. *Immunol. Rev.* **287**, 121–134 (2019).

3. A. J. Thrasher, S. O. Burns, WASP: A key immunological multitasker. *Nat. Rev. Immunol.* **10**, 182–192 (2010).

4. L. M. Machesky, R. H. Insall, Scar1 and the related Wiskott-Aldrich syndrome protein, WASP, regulate the actin cytoskeleton through the Arp2/3 complex. *Curr. Biol.* **8**, 1347–1356 (1998).

5. L. M. Machesky, R. D. Mullins, H. N. Higgs, D. A. Kaiser, L. Blanchoin, R. C. May, M. E. Hall, T. D. Pollard, Scar, a WASp-related protein, activates nucleation of actin filaments by the Arp2/3 complex. *Proc. Natl. Acad. Sci. U.S.A.* **96**, 3739–3744 (1999).

6. S. Tur-Gracia, N. Martinez-Quiles, Emerging functions of cytoskeletal proteins in immune diseases. *J. Cell Sci.* **134**, jcs253534 (2021).

7. K. M. Glaser, J. Doon-Ralls, N. Walters, X. Y. Rima, A. S. Rambold, E. Réategui, T. Lämmermann, Arp2/3 complex and the pentose phosphate pathway regulate late phases of neutrophil swarming. *iScience* **27**, 108656 (2023).

8. E. D. Goley, M. D. Welch, The ARP2/3 complex: An actin nucleator comes of age. *Nat. Rev. Mol. Cell Biol.* **7**, 713–726 (2006).

9. A. M. Gautreau, F. E. Fregoso, G. Simanov, R. Dominguez, Nucleation, stabilization, and disassembly of branched actin networks. *Trends Cell Biol.* **32**, 421–432 (2022).

10. J. V. Abella, C. Galloni, J. Pernier, D. J. Barry, S. Kjær, M.-F. Carlier, M. Way, Isoform diversity in the Arp2/3 complex determines actin filament dynamics. *Nat. Cell Biol.* **18**, 76–86 (2016).

11. C. Galloni, D. Carra, J. V. G. Abella, S. Kjær, P. Singaravelu, D. J. Barry, N. Kogata, C. Guérin, L. Blanchoin, M. Way, MICAL2 enhances branched actin network disassembly by oxidizing Arp3B-containing Arp2/3 complexes. *J. Cell Biol.* **220**, e202102043 (2021).

12. L. Cao, S. Huang, A. Basant, M. Mladenov, M. Way, CK-666 and CK-869 differentially inhibit Arp2/3 iso-complexes. *EMBO Rep.* **25**, 3221–3239 (2024).

13. T. W. Kuijpers, A. T. J. Tool, I. van der Bijl, M. de Boer, M. van Houdt, I. M. de Cuyper, D. Roos, F. van Alphen, K. van Leeuwen, E. L. Cambridge, M. J. Arends, G. Dougan, S. Clare, R. Ramirez-Solis, S. T. Pals, D. J. Adams, A. B. Meijer, T. K. van den Berg, Combined immunodeficiency with severe inflammation and allergy caused by ARPC1B deficiency. *J. Allergy Clin. Immunol.* **140**, 273–277.e10 (2017).

14. W. H. Kahr, F. G. Pluthero, A. Elkadri, N. Warner, M. Drobac, C. H. Chen, R. W. Lo, L. Li, R. Li, Q. Li, C. Thoeni, J. Pan, G. Leung, I. Lara-Corrales, R. Murchie, E. Cutz, R. M. Laxer, J. Upton, C. M. Roifman, R. S. M. Yeung, J. H. Brumell, A. M. Muise, Loss of the

Arp2/3 complex component ARPC1B causes platelet abnormalities and predisposes to inflammatory disease. *Nat. Commun.* **8**, 14816 (2017).

15. L. O. Randzavola, K. Strege, M. Juzans, Y. Asano, J. C. Stinchcombe, C. M. Gawden-Bone, M. N. J. Seaman, T. W. Kuijpers, G. M. Griffiths, Loss of ARPC1B impairs cytotoxic T lymphocyte maintenance and cytolytic activity. *J. Clin. Invest.* **129**, 5600–5614 (2019).
16. S. Volpi, M. P. Cicalese, P. Tuijnenburg, A. T. J. Tool, E. Cuadrado, M. Abu-Halaweh, H. Ahanchian, R. Alzyoud, Z. C. Akdemir, F. Barzaghi, A. Blank, B. Boisson, C. Bottino, I. Brigida, R. Caorsi, J.-L. Casanova, S. Chiesa, I. K. Chinn, G. Dückers, A. Enders, H. C. Erichsen, L. R. Forbes, T. Gambin, M. Gattorno, E. G. Karimiani, S. Giliani, M. S. Gold, E.-M. Jacobsen, M. H. Jansen, J. R. King, R. M. Laxer, J. R. Lupski, E. Mace, S. Marcenaro, R. Maroofian, A. B. Meijer, T. Niehues, L. D. Notarangelo, J. Orange, U. Pannicke, C. Pearson, P. Picco, P. J. Quinn, A. Schulz, F. Seeborg, A. Stray-Pedersen, H. Tawamie, E. M. M. van Leeuwen, A. Aiuti, R. Yeung, K. Schwarz, T. W. Kuijpers, A combined immunodeficiency with severe infections, inflammation, and allergy caused by ARPC1B deficiency. *J. Allergy Clin. Immunol.* **143**, 2296–2299 (2019).
17. C. J. Nunes-Santos, H. Kuehn, B. Boast, S. Hwang, D. B. Kuhns, J. Stoddard, J. E. Niemela, D. L. Fink, S. Pittaluga, M. Abu-Asab, J. S. Davies, V. A. Barr, T. Kawai, O. M. Delmonte, M. Bosticardo, M. Garofalo, M. Carneiro-Sampaio, R. Somech, M. Gharagozlou, N. Parvaneh, L. E. Samelson, T. A. Fleisher, A. Puel, L. D. Notarangelo, B. Boisson, J.-L. Casanova, B. Derfalvi, S. D. Rosenzweig, Inherited ARPC5 mutations cause an actinopathy impairing cell motility and disrupting cytokine signaling. *Nat. Commun.* **14**, 3708 (2023).
18. E. Sindram, A. Caballero-Oteyza, N. Kogata, S. Chor Mei Huang, Z. Alizadeh, L. Gámez-Díaz, M. R. Fazlollhi, X. Peng, B. Grimbacher, M. Way, M. Proietti, ARPC5 deficiency leads to severe early-onset systemic inflammation and mortality. *Dis. Model. Mech.* **16**, dmm050145 (2023).
19. C. L. Abram, G. L. Roberge, Y. Hu, C. A. Lowell, Comparative analysis of the efficiency and specificity of myeloid-Cre deleting strains using ROSA-EYFP reporter mice. *J. Immunol. Methods* **408**, 89–100 (2014).
20. J. Stallhofer, M. Friedrich, A. Konrad-Zerna, M. Wetzke, P. Lohse, J. Glas, C. Tillack-Schreiber, F. Schnitzler, F. Beigel, S. Brand, Lipocalin-2 is a disease activity marker in inflammatory bowel disease regulated by IL-17A, IL-22, and TNF-α and modulated by IL23R genotype status. *Inflamm. Bowel Dis.* **21**, 2327–2340 (2015).
21. X. Li, H. Wang, X. Yu, G. Saha, L. Kalafati, C. Ioannidis, I. Mitroulis, M. G. Netea, T. Chavakis, G. Hajishengallis, Maladaptive innate immune training of myelopoiesis links inflammatory comorbidities. *Cell* **185**, 1709–1727.e18 (2022).
22. Z. Al Nabhani, S. Dulauroy, R. Marques, C. Cousu, S. Al Bounny, F. Déjardin, T. Sparwasser, M. Bérard, N. Cerf-Bensussan, G. Eberl, A weaning reaction to microbiota is required for resistance to immunopathologies in the adult. *Immunity* **50**, 1276–1288.e5 (2019).
23. S. Dikiy, A. Y. Rudensky, Principles of regulatory T cell function. *Immunity* **56**, 240–255 (2023).

24. S. Jin, C. F. Guerrero-Juarez, L. Zhang, I. Chang, R. Ramos, C.-H. Kuan, P. Myung, M. V. Plikus, Q. Nie, Inference and analysis of cell-cell communication using CellChat. *Nat. Commun.* **12**, 1088 (2021).

25. W. J. Branchett, M. Saraiva, A. O'Garra, Regulation of inflammation by Interleukin-10 in the intestinal and respiratory mucosa. *Curr. Opin. Immunol.* **91**, 102495 (2024).

26. D. Moratto, S. Giliani, C. Bonfim, E. Mazzolari, A. Fischer, H. D. Ochs, A. J. Cant, A. J. Thrasher, M. J. Cowan, M. H. Albert, T. Small, S.-Y. Pai, E. Haddad, A. Lisa, S. Hambleton, M. Slatter, M. Cavazzana-Calvo, N. Mahlaoui, C. Picard, T. R. Torgerson, L. Burroughs, A. Koliski, J. Z. Neto, F. Porta, W. Qasim, P. Veys, K. Kavanau, M. Hönig, A. Schulz, W. Friedrich, L. D. Notarangelo, Long-term outcome and lineage-specific chimerism in 194 patients with Wiskott-Aldrich syndrome treated by hematopoietic cell transplantation in the period 1980-2009: An international collaborative study. *Blood* **118**, 1675–1684 (2011).

27. L. M. Burroughs, A. Petrovic, R. Brazauskas, X. Liu, L. M. Griffith, H. D. Ochs, J. J. Bleesing, S. Edwards, C. C. Dvorak, S. Chaudhury, S. E. Prockop, R. Quinones, F. D. Goldman, T. C. Quigg, S. Chandrakasan, A. R. Smith, S. Parikh, B. J. Dávila Saldaña, M. S. Thakar, R. Phelan, S. Shenoy, L. R. Forbes, C. Martinez, D. Chellapandian, E. Shereck, H. K. Miller, N. Kapoor, J. L. Barnum, H. Chong, D. C. Shyr, K. Chen, R. Abu-Arja, A. J. Shah, K. G. Weinacht, T. B. Moore, A. Joshi, K. B. DeSantes, A. P. Gillio, G. D. E. Cuvelier, M. D. Keller, J. Rozmus, T. Torgerson, M. A. Pulsipher, E. Haddad, K. E. Sullivan, B. R. Logan, D. B. Kohn, J. M. Puck, L. D. Notarangelo, S.-Y. Pai, D. J. Rawlings, M. J. Cowan, Excellent outcomes following hematopoietic cell transplantation for Wiskott-Aldrich syndrome: A PIDTC report. *Blood* **135**, 2094–2105 (2020).

28. N. Morita, E. Umemoto, S. Fujita, A. Hayashi, J. Kikuta, I. Kimura, T. Haneda, T. Imai, A. Inoue, H. Mimuro, Y. Maeda, H. Kayama, R. Okumura, J. Aoki, N. Okada, T. Kida, M. Ishii, R. Nabeshima, K. Takeda, GPR31-dependent dendrite protrusion of intestinal $CX3CR1^+$ cells by bacterial metabolites. *Nature* **566**, 110–114 (2019).

29. M. Kim, C. Galan, A. A. Hill, W.-J. Wu, H. Fehlner-Peach, H. W. Song, D. Schady, M. L. Bettini, K. W. Simpson, R. S. Longman, D. R. Littman, G. E. Diehl, Critical role for the microbiota in CX_3CR1^+ intestinal mononuclear phagocyte regulation of intestinal T cell responses. *Immunity* **49**, 151–163.e5 (2018).

30. A. Mortha, A. Chudnovskiy, D. Hashimoto, M. Bogunovic, S. P. Spencer, Y. Belkaid, M. Merad, Microbiota-dependent crosstalk between macrophages and ILC3 promotes intestinal homeostasis. *Science* **343**, 1249288 (2014).

31. G. E. Diehl, R. S. Longman, J.-X. Zhang, B. Breart, C. Galan, A. Cuesta, S. R. Schwab, D. R. Littman, Microbiota restricts trafficking of bacteria to mesenteric lymph nodes by CX_3CR1^{hi} cells. *Nature* **494**, 116–120 (2013).

32. Y. R. Na, M. Stakenborg, S. H. Seok, G. Matteoli, Macrophages in intestinal inflammation and resolution: A potential therapeutic target in IBD. *Nat. Rev. Gastroenterol. Hepatol.* **16**, 531–543 (2019).

33. L. Jostins, S. Ripke, R. K. Weersma, R. H. Duerr, D. P. McGovern, K. Y. Hui, J. C. Lee, L. P. Schumm, Y. Sharma, C. A. Anderson, J. Essers, M. Mitrovic, K. Ning, I. Cleynen, E. Theatre, S. L. Spain, S. Raychaudhuri, P. Goyette, Z. Wei, C. Abraham, J.-P. Achkar, T.

Ahmad, L. Amininejad, A. N. Ananthakrishnan, V. Andersen, J. M. Andrews, L. Baidoo, T. Balschun, P. A. Bampton, A. Bitton, G. Boucher, S. Brand, C. Büning, A. Cohain, S. Cichon, M. D'Amato, D. De Jong, K. L. Devaney, M. Dubinsky, C. Edwards, D. Ellinghaus, L. R. Ferguson, D. Franchimont, K. Fransen, R. Gearry, M. Georges, C. Gieger, J. Glas, T. Haritunians, A. Hart, C. Hawkey, M. Hedl, X. Hu, T. H. Karlsen, L. Kupcinskas, S. Kugathasan, A. Latiano, D. Laukens, I. C. Lawrance, C. W. Lees, E. Louis, G. Mahy, J. Mansfield, A. R. Morgan, C. Mowat, W. Newman, O. Palmieri, C. Y. Ponsioen, U. Potocnik, N. J. Prescott, M. Regueiro, J. I. Rotter, R. K. Russell, J. D. Sanderson, M. Sans, J. Satsangi, S. Schreiber, L. A. Simms, J. Sventoraityte, S. R. Targan, K. D. Taylor, M. Tremelling, H. W. Verspaget, M. De Vos, C. Wijmenga, D. C. Wilson, J. Winkelmann, R. J. Xavier, S. Zeissig, B. Zhang, C. K. Zhang, H. Zhao, M. S. Silverberg, V. Annese, H. Hakonarson, S. R. Brant, G. Radford-Smith, C. G. Mathew, J. D. Rioux, E. E. Schadt, M. J. Daly, A. Franke, M. Parkes, S. Vermeire, J. C. Barrett, J. H. Cho, International IBD Genetics Consortium (IIBDGC), Host-microbe interactions have shaped the genetic architecture of inflammatory bowel disease. *Nature* **491**, 119–124 (2012).

34. M. Bolger-Munro, K. Choi, J. M. Scurll, L. Abraham, R. S. Chappell, D. Sheen, M. Dang-Lawson, X. Wu, J. J. Priatel, D. Coombs, J. A. Hammer, M. R. Gold, Arp2/3 complex-driven spatial patterning of the BCR enhances immune synapse formation, BCR signaling and B cell activation. *eLife* **8**, e44574 (2019).

35. R. C. May, E. Caron, A. Hall, L. M. Machesky, Involvement of the Arp2/3 complex in phagocytosis mediated by FcgammaR or CR3. *Nat. Cell Biol.* **2**, 246–248 (2000).

36. S. A. Cook, W. A. Comrie, M. C. Poli, M. Similuk, A. J. Oler, A. J. Faruqi, D. B. Kuhns, S. Yang, A. Vargas-Hernández, A. F. Carisey, B. Fournier, D. E. Anderson, S. Price, M. Smelkinson, W. Abou Chahla, L. R. Forbes, E. M. Mace, T. N. Cao, Z. H. Coban-Akdemir, S. N. Jhangiani, D. M. Muzny, R. A. Gibbs, J. R. Lupski, J. S. Orange, G. D. E. Cuvelier, M. Al Hassani, N. Al Kaabi, Z. Al Yafei, S. Jyonouchi, N. Raje, J. W. Caldwell, Y. Huang, J. K. Burkhardt, S. Latour, B. Chen, G. ElGhazali, V. K. Rao, I. K. Chinn, M. J. Lenardo, HEM1 deficiency disrupts mTORC2 and F-actin control in inherited immunodysregulatory disease. *Science* **369**, 202–207 (2020).

37. A. J. Thrasher, New insights into the biology of Wiskott-Aldrich syndrome (WAS). *Hematology Am. Soc. Hematol. Educ. Program* **2009**, 132–138 (2009).

38. D. D. Nguyen, S. Muthupalani, J. A. Goettel, M. A. Eston, M. Mobley, N. S. Taylor, A. McCabe, R. Marin, S. B. Snapper, J. G. Fox, Colitis and colon cancer in WASP-deficient mice require helicobacter species. *Inflamm. Bowel Dis.* **19**, 2041–2050 (2013).

39. D. D. Nguyen, M.-A. Wurbel, J. A. Goettel, M. A. Eston, O. S. Ahmed, R. Marin, E. K. Boden, E. J. Villablanca, H. Paidassi, V. Ahuja, H.-C. Reinecker, E. Fiebiger, A. Lacy-Hulbert, B. H. Horwitz, J. R. Mora, S. B. Snapper, Wiskott-Aldrich syndrome protein deficiency in innate immune cells leads to mucosal immune dysregulation and colitis in mice. *Gastroenterology* **143**, 719–729.e2 (2012).

40. M. E. Johansson, M. Phillipson, J. Petersson, A. Velcich, L. Holm, G. C. Hansson, The inner of the two Muc2 mucin-dependent mucus layers in colon is devoid of bacteria. *Proc. Natl. Acad. Sci. U.S.A.* **105**, 15064–15069 (2008).

41. M. Van der Sluis, B. A. E. De Koning, A. C. J. M. De Bruijn, A. Velcich, J. P. P. Meijerink, J. B. Van Goudoever, H. A. Büller, J. Dekker, I. Van Seuningen, I. B. Renes, A. W. C. Einerhand, Muc2-deficient mice spontaneously develop colitis, indicating that MUC2 is critical for colonic protection. *Gastroenterology* **131**, 117–129 (2006).

42. C. A. Rivera, V. Randrian, W. Richer, Y. Gerber-Ferder, M.-G. Delgado, A. S. Chikina, A. Frede, C. Sorini, M. Maurin, H. Kammoun-Chaari, S. M. Parigi, C. Goudot, M. Cabeza-Cabrerizo, S. Baulande, S. Lameiras, P. Guermonprez, C. Reis e Sousa, M. Lecuit, H. D. Moreau, J. Helft, D. M. Vignjevic, E. J. Villablanca, A.-M. Lennon-Duménil, Epithelial colonization by gut dendritic cells promotes their functional diversification. *Immunity* **55**, 129–144.e8 (2022).

43. P. Essletzbichler, V. Sedlyarov, F. Frommelt, D. Soulat, L. X. Heinz, A. Stefanovic, B. Neumayer, G. Superti-Furga, A genome-wide CRISPR functional survey of the human phagocytosis molecular machinery. *Life Sci. Alliance* **6**, e202201715 (2023).

44. A. Wiedemann, S. Linder, G. Grassl, M. Albert, I. Autenrieth, M. Aepfelbacher, Yersinia enterocolitica invasin triggers phagocytosis via beta1 integrins, CDC42Hs and WASp in macrophages. *Cell. Microbiol.* **3**, 693–702 (2001).

45. M. Krendel, N. C. Gauthier, Building the phagocytic cup on an actin scaffold. *Curr. Opin. Cell Biol.* **77**, 102112 (2022).

46. K. Gettler, R. Levantovsky, A. Moscati, M. Giri, Y. Wu, N.-Y. Hsu, L.-S. Chuang, A. Sazonovs, S. Venkateswaran, U. Korie, C. Chasteau, R. H. Duerr, M. S. Silverberg, S. B. Snapper, M. J. Daly, D. P. McGovern, S. R. Brant, J. D. Rioux, S. Kugathasan, C. A. Anderson, Y. Itan, J. H. Cho, UK IBD Genetics Consortium, National Institute of Diabetes, Digestive and Kidney Diseases Inflammatory Bowel Disease Genetics Consortium, Common and rare variant prediction and penetrance of IBD in a large, multi-ethnic, health system-based biobank cohort. *Gastroenterology* **160**, 1546–1557 (2021).

47. C. Bolton, C. S. Smillie, S. Pandey, R. Elmentaite, G. Wei, C. Argmann, D. Aschenbrenner, K. R. James, D. P. B. McGovern, M. Macchi, J. Cho, D. S. Shouval, J. Kammermeier, S. Koletzko, K. Bagalopal, M. Capitani, A. Cavounidis, E. Pires, C. Weidinger, J. McCullagh, P. D. Arkwright, W. Haller, B. Siegmund, L. Peters, L. Jostins, S. P. L. Travis, C. A. Anderson, S. Snapper, C. Klein, E. Schadt, M. Zilbauer, R. Xavier, S. Teichmann, A. M. Muise, A. Regev, H. H. Uhlig, An integrated taxonomy for monogenic inflammatory bowel disease. *Gastroenterology* **162**, 859–876 (2022).

48. J. Riedl, A. H. Crevenna, K. Kessenbrock, J. H. Yu, D. Neukirchen, M. Bista, F. Bradke, D. Jenne, T. A. Holak, Z. Werb, M. Sixt, R. Wedlich-Soldner, Lifeact: A versatile marker to visualize F-actin. *Nat. Methods* **5**, 605–607 (2008).

49. P. J. Koelink, M. E. Wildenberg, L. W. Stitt, B. G. Feagan, M. Koldijk, A. B. van 't Wout, R. Atreya, M. Vieth, J. F. Brandse, S. Duijst, A. A. Te Velde, G. R. A. M. D'Haens, B. G. Levesque, G. R. van den Brink, Development of reliable, valid and responsive scoring systems for endoscopy and histology in animal models for inflammatory bowel disease. *J. Crohns Colitis* **12**, 794–803 (2018).

50. H. Aegerter, J. Kulikauskaite, S. Crotta, H. Patel, G. Kelly, E. M. Hessel, M. Mack, S. Beinke, A. Wack, Influenza-induced monocyte-derived alveolar macrophages confer prolonged antibacterial protection. *Nat. Immunol.* **21**, 145–157 (2020).
